# Supplementary material for: Direct Interactions with Nascent Transcripts Is Potentially a Common Targeting Mechanism of Long Non-Coding RNAs
Source: Genes (Basel). 2020 Dec 10;11(12):1483. doi: 10.3390/genes11121483 (PMC7764144; doi:10.3390/genes11121483)

All Chromosomes

Query population : 2667
Reference population : 1243
Relative Ks p−value : 1.43985e−12
Relative ecdf deviation area : 0.0215167
Relative ecdf area correlation : 0.0862894
Relative ecdf deviation area p−value : <0.01
Scaled Absolute min. distance p−value : <0.01
Scaled Absolute min. lower tail : TRUE
Jaccard Measure p−value : 0.1
Jaccard Measure lower tail : FALSE
Projection test p−value : 0.00388599
Projection test lower tail : FALSE
Projection test observed to expected ratio : 11.0042

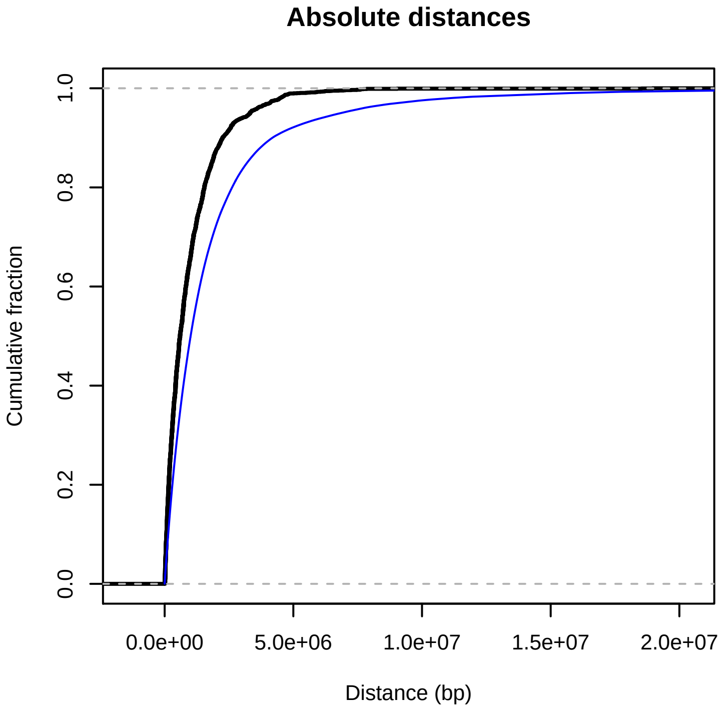

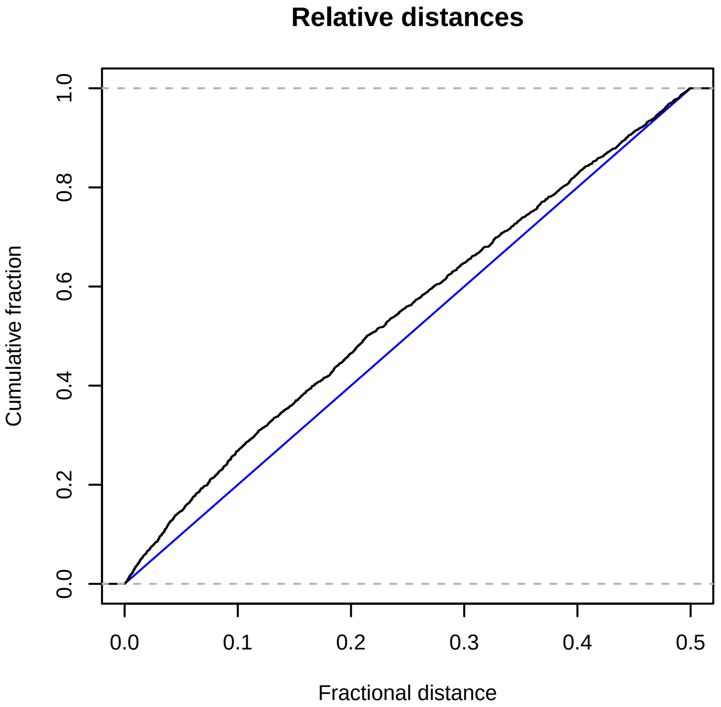

Supplement: Supplementary file 1 [file genes-11-01483-s001.zip › Supplementary Data S1/Supplementary Data/images/other/margi_genometric.AD_07.pdf]
