# Supplementary material for: Direct Interactions with Nascent Transcripts Is Potentially a Common Targeting Mechanism of Long Non-Coding RNAs
Source: Genes (Basel). 2020 Dec 10;11(12):1483. doi: 10.3390/genes11121483 (PMC7764144; doi:10.3390/genes11121483)

All Chromosomes

Query population : 1668
Reference population : 1243
Relative Ks p−value : 9.52247e−10
Relative ecdf deviation area : 0.0245289
Relative ecdf area correlation : 0.0986966
Relative ecdf deviation area p−value : <0.01
Scaled Absolute min. distance p−value : <0.01
Scaled Absolute min. lower tail : TRUE
Jaccard Measure p−value : 0.41
Jaccard Measure lower tail : TRUE
Projection test p−value : 0.055251
Projection test lower tail : FALSE
Projection test observed to expected ratio : 0

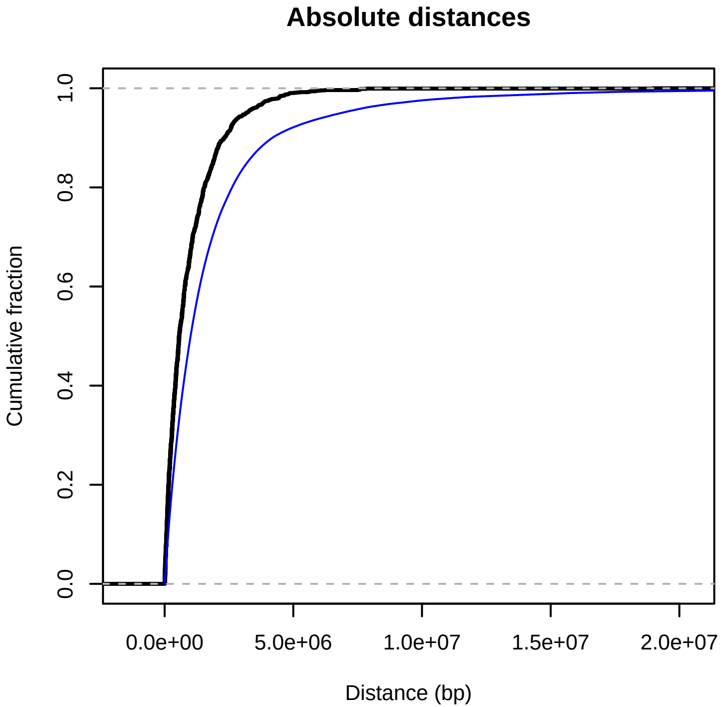

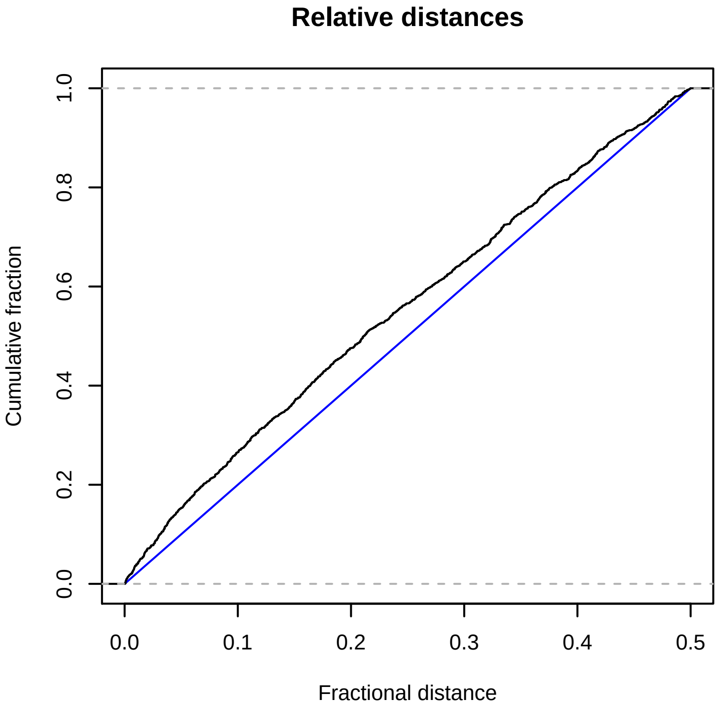

Supplement: Supplementary file 1 [file genes-11-01483-s001.zip › Supplementary Data S1/Supplementary Data/images/other/margi_genometric.AD_10.pdf]
