# Supplementary material for: Direct Interactions with Nascent Transcripts Is Potentially a Common Targeting Mechanism of Long Non-Coding RNAs
Source: Genes (Basel). 2020 Dec 10;11(12):1483. doi: 10.3390/genes11121483 (PMC7764144; doi:10.3390/genes11121483)
Supplement: Supplementary file 1 [file genes-11-01483-s001.zip › Supplementary Data S1/Supplementary Data/images/two_pvalues_post.pdf]

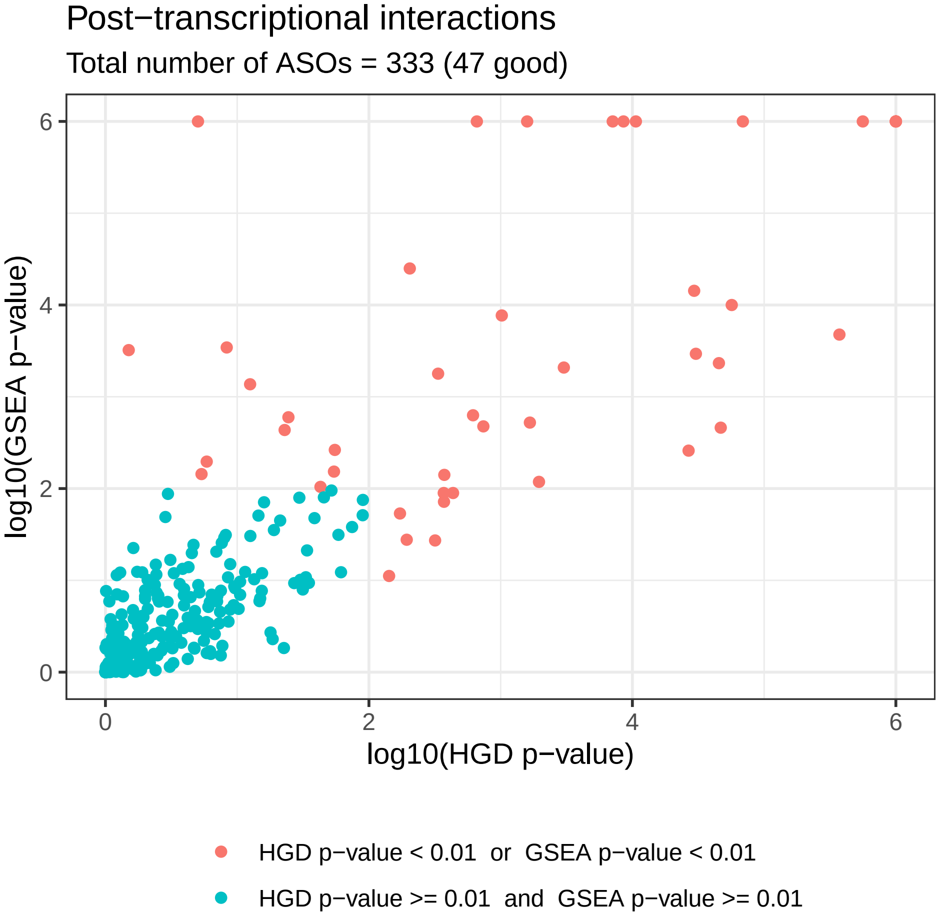
Post−transcriptional interactions
Total number of ASOs = 333 (47 good)
log10(GSEA p−value)
log10(HGD p−value)
0
2
4
6
0
2
4
6
HGD p−value < 0.01 or GSEA p−value < 0.01
HGD p−value >= 0.01 and GSEA p−value >= 0.01
