# Supplementary material for: Direct Interactions with Nascent Transcripts Is Potentially a Common Targeting Mechanism of Long Non-Coding RNAs
Source: Genes (Basel). 2020 Dec 10;11(12):1483. doi: 10.3390/genes11121483 (PMC7764144; doi:10.3390/genes11121483)
Supplement: Supplementary file 1 [file genes-11-01483-s001.zip › Supplementary Data S1/Supplementary Data/images/workflow.pptx]

## Slide 1
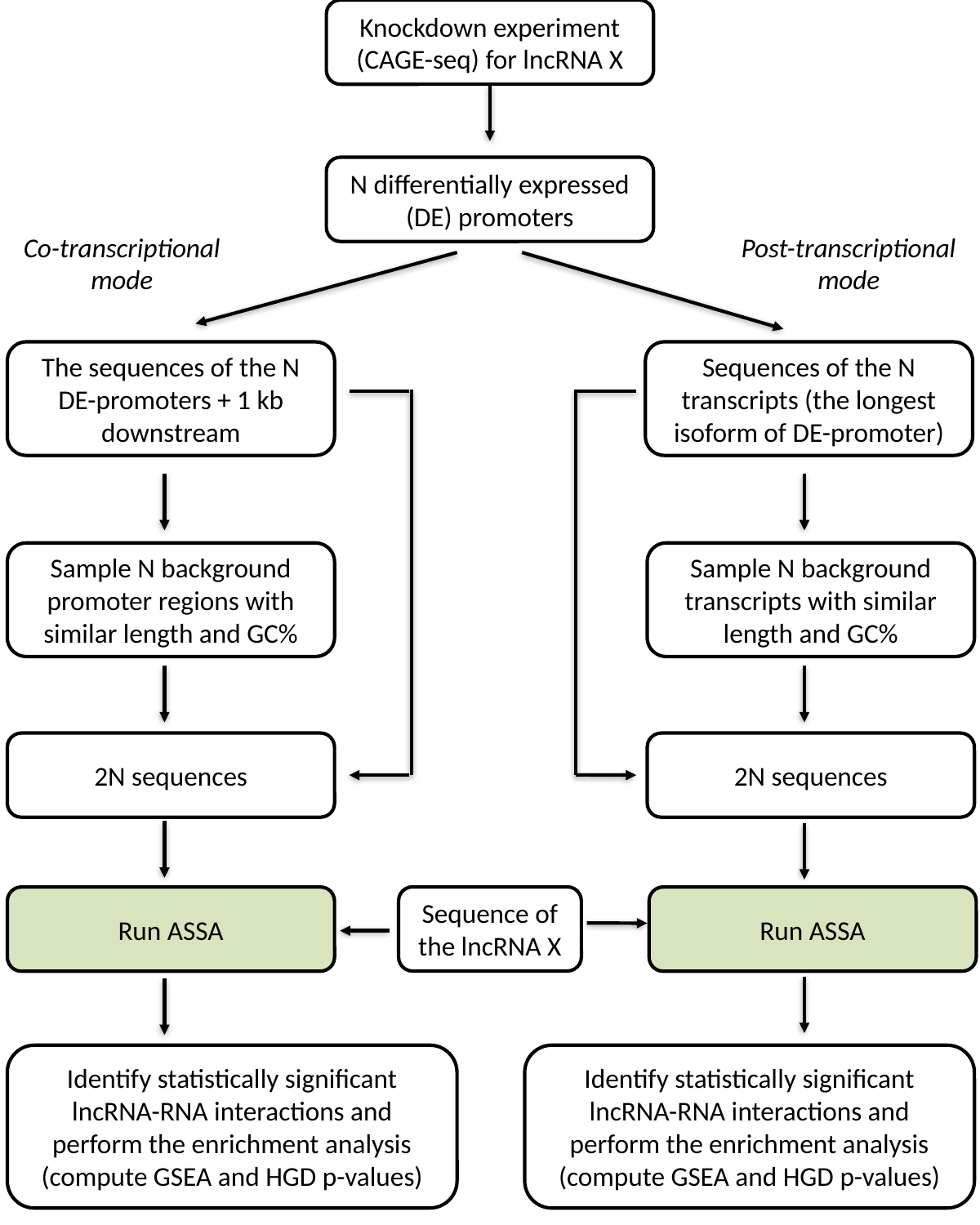

Knockdown experiment (CAGE-seq) for lncRNA X
N differentially expressed (DE) promoters
Co-transcriptional mode
Post-transcriptional mode
The sequences of the N DE-promoters + 1 kb downstream
Sequences of the N transcripts (the longest isoform of DE-promoter)
Sample N background promoter regions with similar length and GC%
Sample N background transcripts with similar length and GC%
2N sequences
2N sequences
Sequence of the lncRNA X
Run ASSA
Run ASSA
Identify statistically significant lncRNA-RNA interactions and perform the enrichment analysis (compute GSEA and HGD p-values)
Identify statistically significant lncRNA-RNA interactions and perform the enrichment analysis (compute GSEA and HGD p-values)
