# Supplementary material for: Direct Interactions with Nascent Transcripts Is Potentially a Common Targeting Mechanism of Long Non-Coding RNAs
Source: Genes (Basel). 2020 Dec 10;11(12):1483. doi: 10.3390/genes11121483 (PMC7764144; doi:10.3390/genes11121483)
Supplement: Supplementary file 1 [file genes-11-01483-s001.zip › Supplementary Figures.pdf]

341 **Supplementary figures**

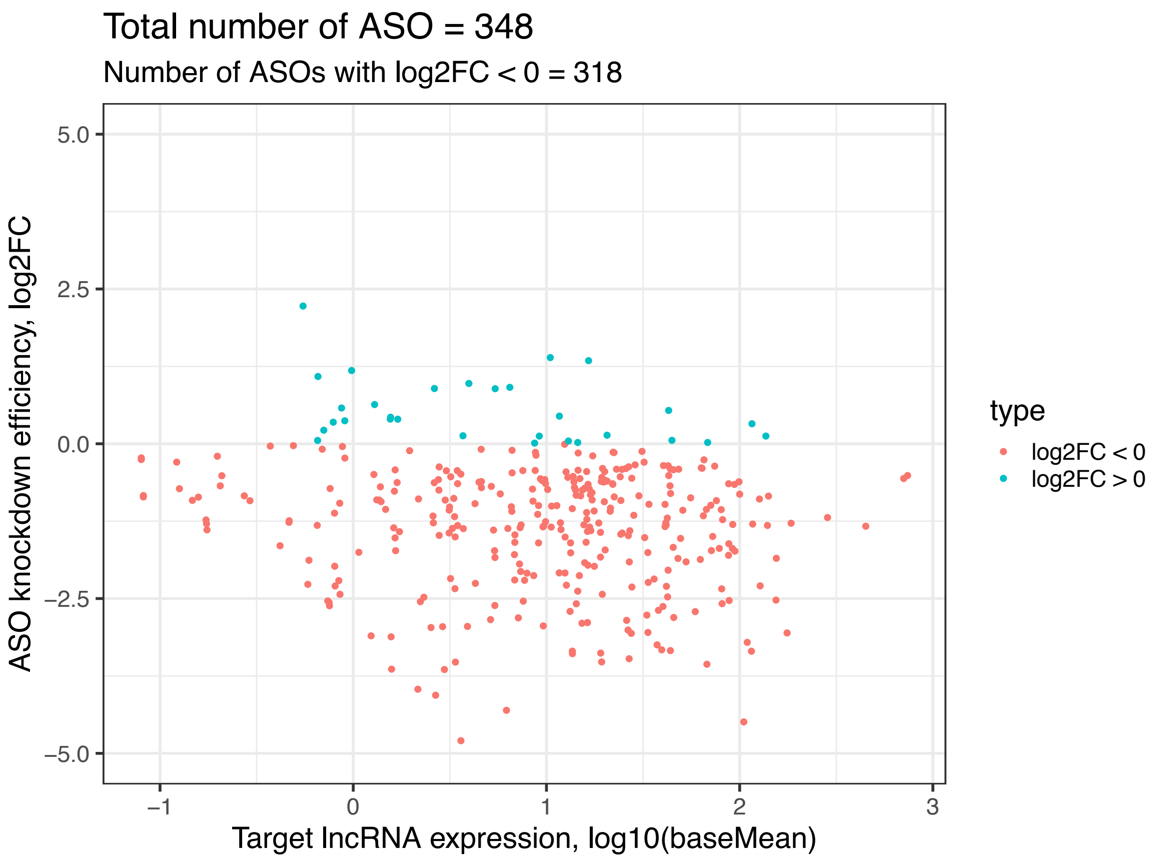

Supplementary Figure S1. Efficiency of all the ASO knockdowns in the FANTOM6 project.

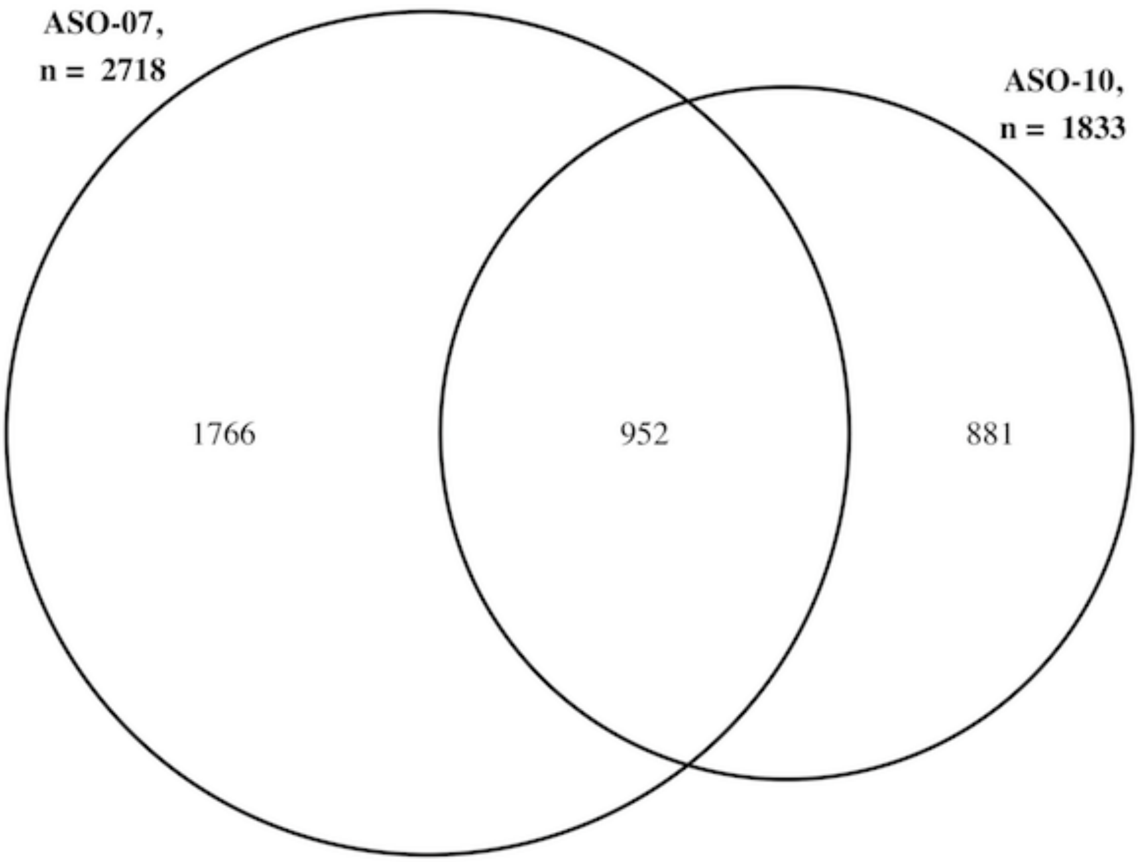

Supplementary Figure S2. The overlap between differentially expressed genes identified in two independent knockdown experiments of the *CHASERR* lncRNA.

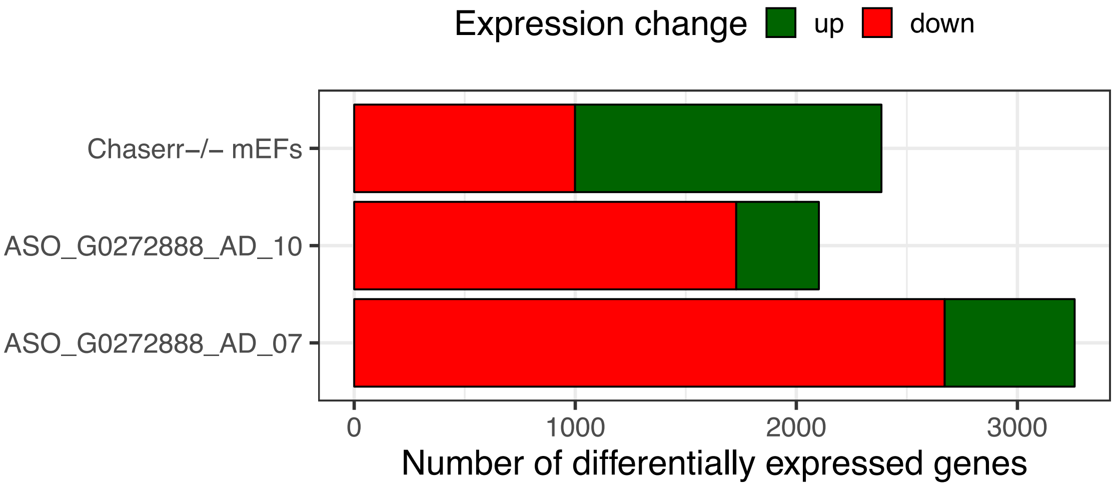

Supplementary Figure S3. Types of differentially expressed genes in two independent *CHASERR* knockdown experiments and in *Chaserr*-/- mouse embryonic fibroblases (mEFs).

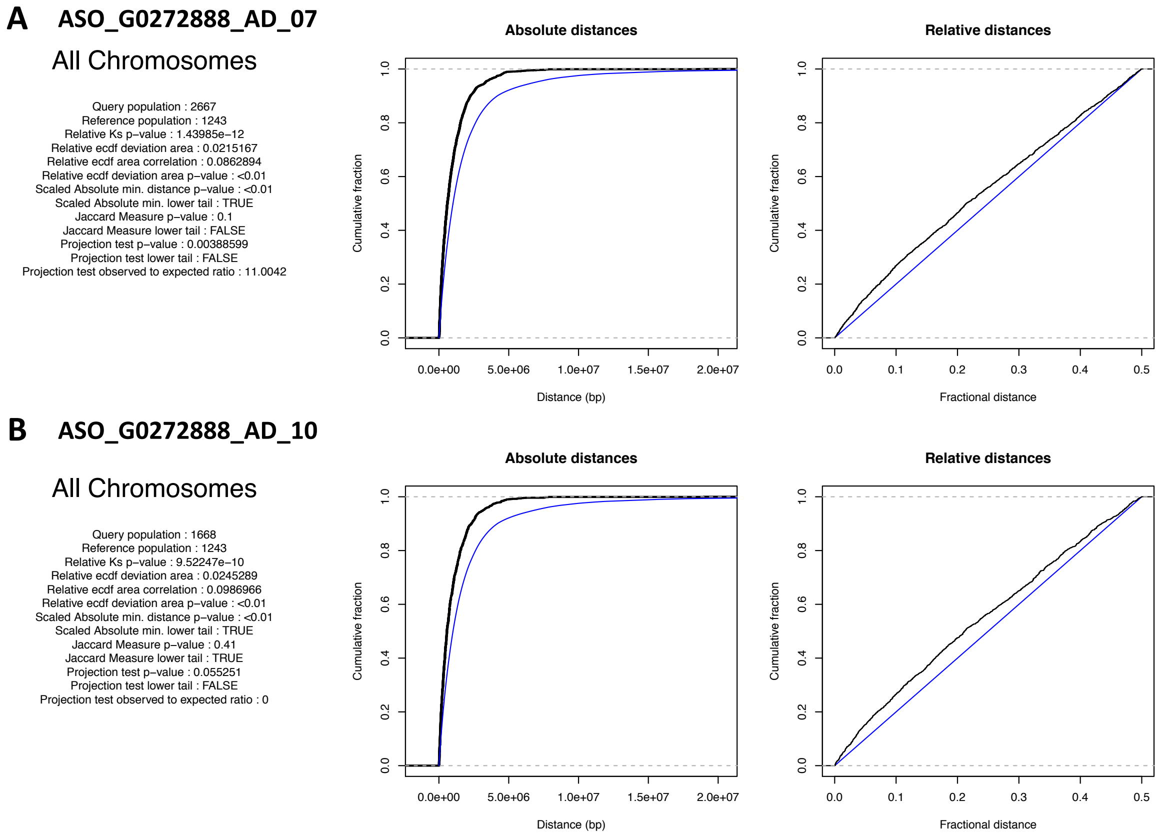

Supplementary Figure S4. Output of the GenometriCorr tool for the two ASOs corresponding to the lncRNA AC013394.2 (*CHASERR*).

```
CLUSTAL multiple sequence alignment by MUSCLE (3.8)


ENSMUST00000184554.7      ------------------------------------------------------------
ENST00000556895.T0        CACCCCGAGCTGTCAGGCGTTCCGCCGCGGCCGCGAGGCCCGCCGGCCGGCGGGGAGCTA


ENSMUST00000184554.7      ------------------------------------------------------------
ENST00000556895.T0        CGCCCGGACGGCCAGCAGGCCCGCGGGAGTGGGGCTGCCGCGGCTGAGGCGAGGCGGGCC


ENSMUST00000184554.7      -CGCGCG-CGATGTGGGAGCTCGCGGCCGGAGCGCCCGGGGAGGCCGGGCCCACGACGCC
ENST00000556895.T0        GCGCGCGTCGGCGTCACAGCCCGCGGCAGAGGCGCCCAGGGCGGCCGGGCCCACGACGCC
                           ****** **  **   *** ****** *  ****** *** ******************

ENSMUST00000184554.7      CGTGGCTCCGCTGCGGCAGCGGCGGTGCTGGTGTCGCGCGCGGCCGGGAGGCGGCTTCGC
ENST00000556895.T0        GAAAGCGCCGCTGCGGTTGCCGCCTCGGAGGC-TCCCCCGGGCCCCGGCGGCTGGACC-C
                              ** *********  ** **   *  **  ** * ** * ** ** *** *   * *

ENSMUST00000184554.7      GCCGCGGGCGGGAGGCTGCGGCGGGCGACCCGTCCTGGACACGCGAGGAAGAGCGAGCCG
ENST00000556895.T0        GGCGCGGGCGGGAGGCTCGGGCGGGCGGTCCGGCCCGGG-ACTCGGGTTTGGGCGACCAG
                          * ***************  ********  *** ** **  ** ** *   * **** * *

ENSMUST00000184554.7      ATGGCGGCAGGGGCCGCGCTTCGACCCGGTAACTTAGAAGATGATAATTAATGTGGTTGC
ENST00000556895.T0        GAGGTGCCGGTGGCCGCGCTCGGACCCGGTGACTTAAGAGATTAAAATTAATTTGGTTGC
                            ** * * * *********  ******** *****  **** * ******* *******

ENSMUST00000184554.7      TGATAATTCTGAATAAATACAG----CTTTTATCCCAGGTGTGCCATTTTGAAGACTGAG
ENST00000556895.T0        TGTTGGTTCTGAACAAATAATGAGTTCTTTTATTTGAGGTATGCCATTTTGAAGACTGAG
                          ** *  ******* *****  *    *******   **** *******************

ENSMUST00000184554.7      ACCATAGAGTTCTA------AGAATAAAGGAAAGAGCCCTTGGGAA----ATTATTATAT
ENST00000556895.T0        ACGTTGGAGTTTTATCCTAGAGGATAAAGGAAA---TCTTTGGGAAAGTCAGTATTTTAT
                          **  * ***** **      ** **********    * *******    * **** ***

ENSMUST00000184554.7      ATAGCAAAAATGTGAATCCTCAGATGGAA-----TGAAAGGCCTGCACCA-----TAGAC
ENST00000556895.T0        ATAGCAAAAATATGAA-CCTCAAACTGAATCCTCTAAAGGCCCCATACAATGAAGTAGAC
                          *********** **** ***** *  ***     * ** * **   ** *     *****

ENSMUST00000184554.7      ATC----GAAGCATTTAAATTTTTTTCTTCTAATTTTTTATGAAGCACCCCGCTTGAAGA
ENST00000556895.T0        ATTGGTGGAAACATTTACACC------------------------CAGTCAGCTTGAAGA
                          **     *** ****** *                          **  * *********

ENSMUST00000184554.7      GTTTGAAATGGACTTTACCACTGAGAAATCAAGATGGCAGCCCATTATGGGGAATTGAGG
ENST00000556895.T0        GTTTCAAATGGACCCTACCACTGAGAAATCAAGATGGCAGTCTACTATGGGGAATTGAGG
                          **** ********  ************************* * * ***************

ENSMUST00000184554.7      -AAAATGGATTAATGCAAGAATGCTGTAATATTATACAACCAACACAGGATTCTTTTAAT
ENST00000556895.T0        AAAAATGGATTAATACAAGAGTCCTGTGATAATATACAACCAAGACAGGGTTCTTTTAAC
                           ************* ***** * **** *** *********** ***** *********

ENSMUST00000184554.7      GTGGATTCCATGAAATGAATGA----------TTCTTACCCAACACAAATGGACAGTGGA
ENST00000556895.T0        ATGGATTCCATGCAGTGAATGAAGACATAGGTTTCTTACCCAACACAAATGGACAGTGGA
                           *********** * *******          ****************************

ENSMUST00000184554.7      ATTTACTTCCTAAAGACTTGTTACATGTCATGTACATTTTTGACATCTGGAGAAGACTCT
ENST00000556895.T0        TTTGACTTTCTAAAGACTTTTT-----TTGTGGACA-TTTTGACATTTGGAGAAG-----
                           ** **** ********** **     *  ** *** ********* ********

ENSMUST00000184554.7      ACAATTCTACAAATGGTAGTTTGTATTCCTGGAATTTCTTGCAGTTTGATCTGAAGTGAC
ENST00000556895.T0        ------------------------------------------------------------


ENSMUST00000184554.7      CTTATGGAATGTTAACTTTAATAAAATCTCTAAAACTTAAAAA
ENST00000556895.T0        ---------------------------------------AAAG
                                                                 ***
```

Supplementary Figure S5. Pairwise sequence alignment of the human and mouse *CHASERR* orthologs.

© 2020 by the authors. Submitted to *Genes* for possible open access publication under the terms and conditions of the Creative Commons Attribution (CC BY) license (http://creativecommons.org/licenses/by/4.0/).
