# Supplementary figures and images for: Direct Interactions with Nascent Transcripts Is Potentially a Common Targeting Mechanism of Long Non-Coding RNAs
Source: Genes (Basel). 2020 Dec 10;11(12):1483. doi: 10.3390/genes11121483 (PMC7764144; doi:10.3390/genes11121483)

Total number of ASO = 348

Number of ASOs with  $\log_2\text{FC} < 0$  = 318

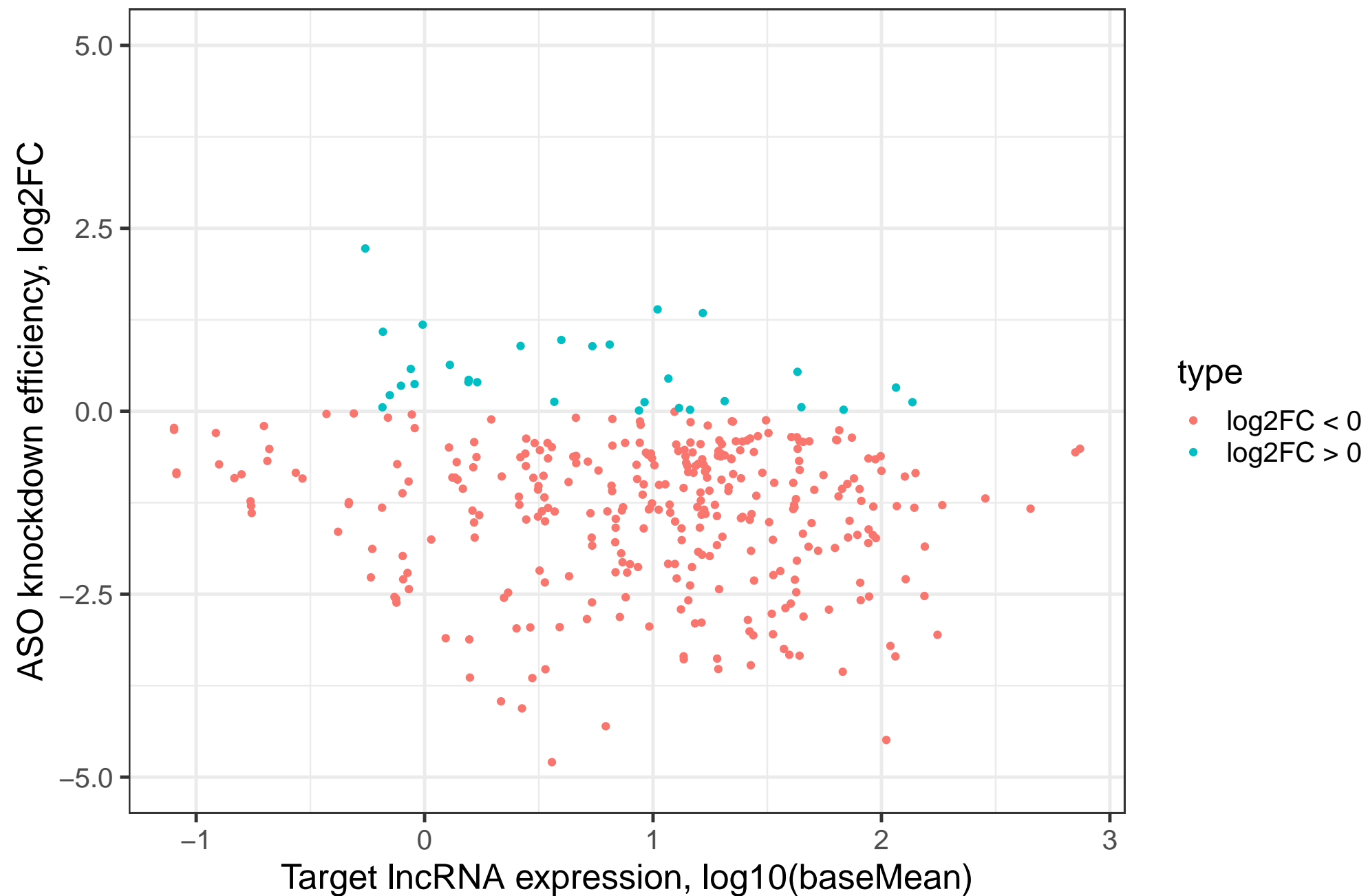

Supplement: Supplementary file 1 [file genes-11-01483-s001.zip › Supplementary Data S1/Supplementary Data/images/ASO_efficiency.pdf]

# 98 good ASOs

ASSA pvalue < 0.01

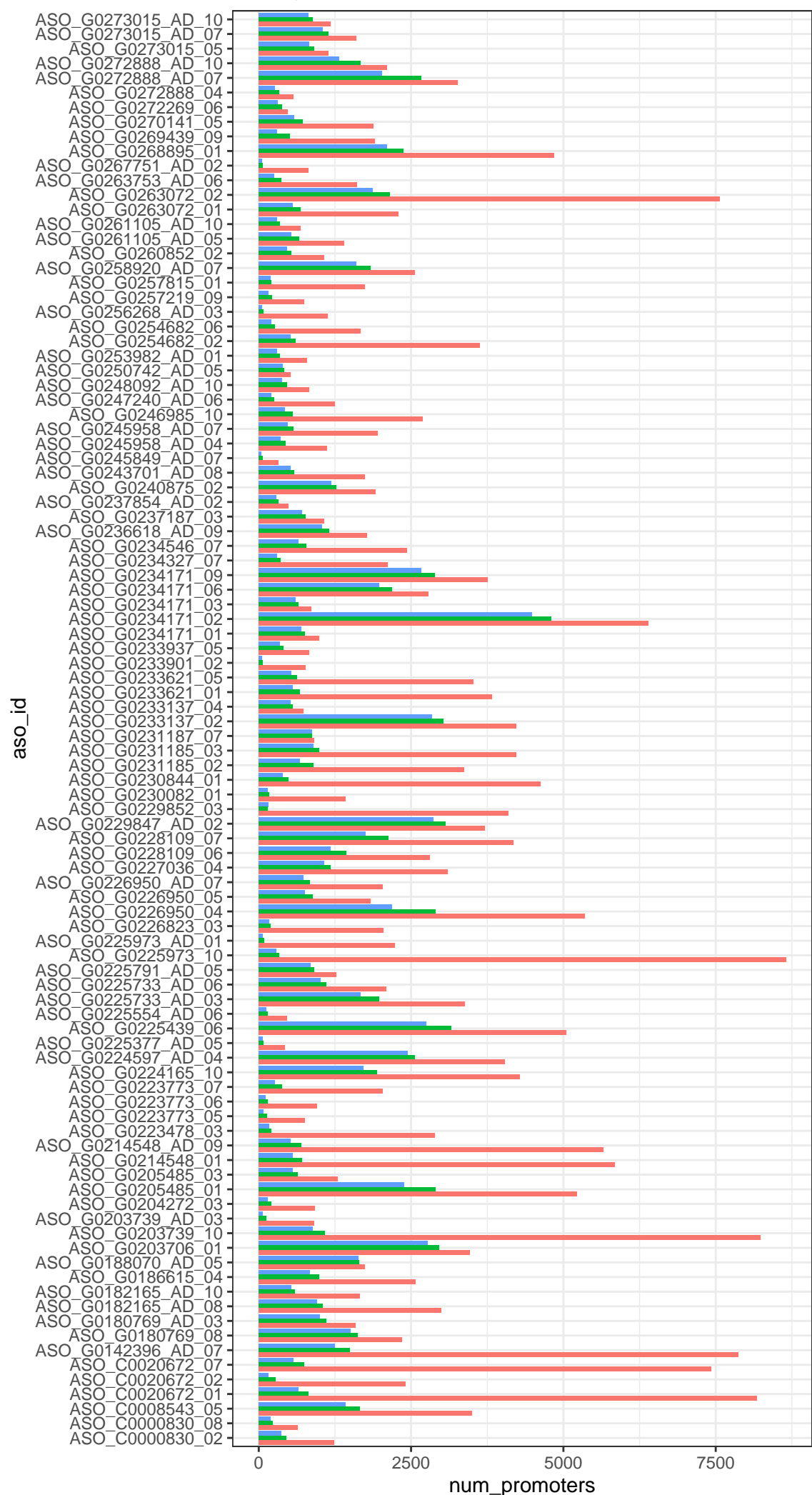

group de\_trxs de\_and\_assa\_hit non\_de\_and\_assa\_hit

Supplement: Supplementary file 1 [file genes-11-01483-s001.zip › Supplementary Data S1/Supplementary Data/images/assa_hits_among_de_and_non_de.CO.pdf]

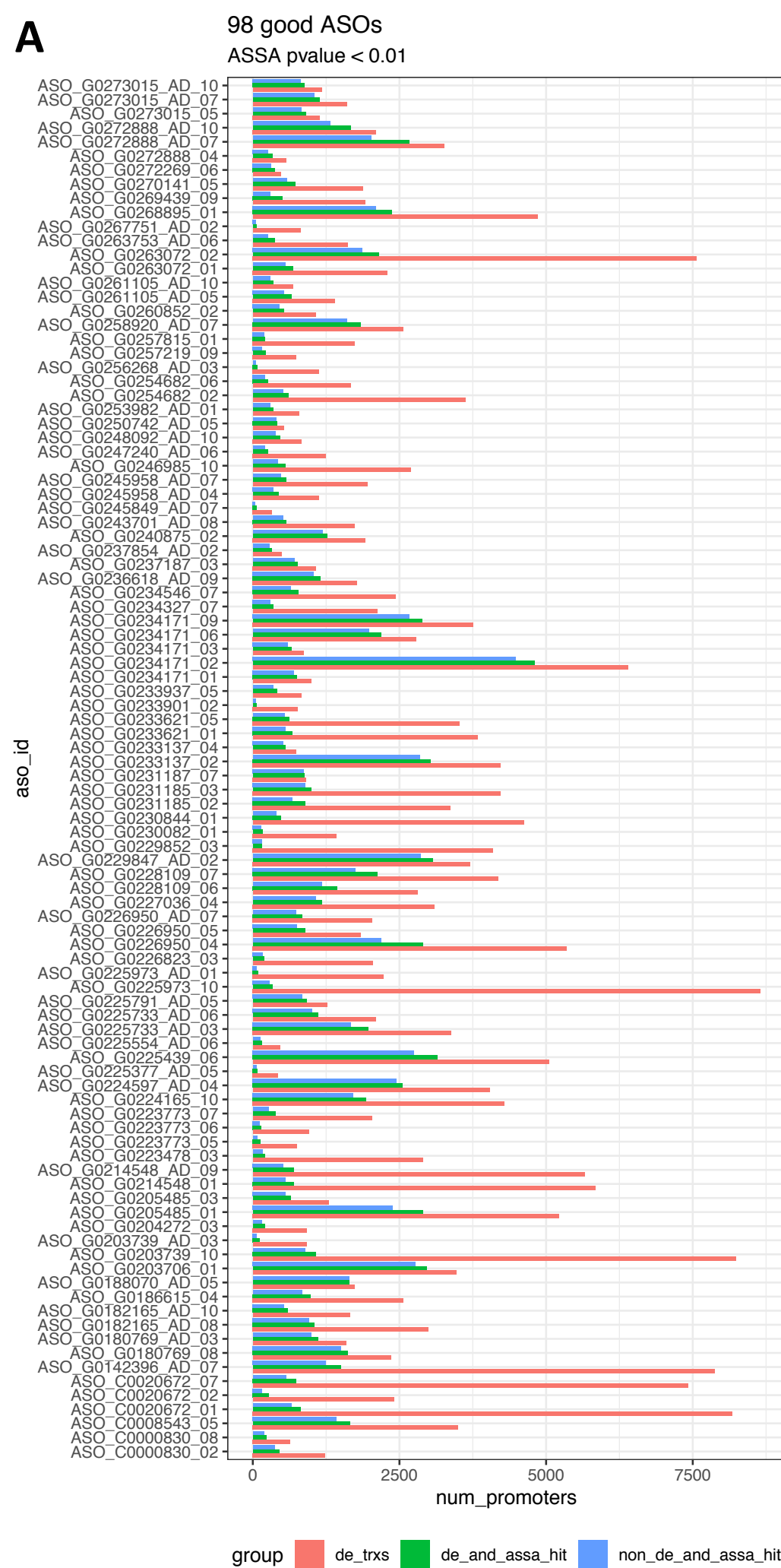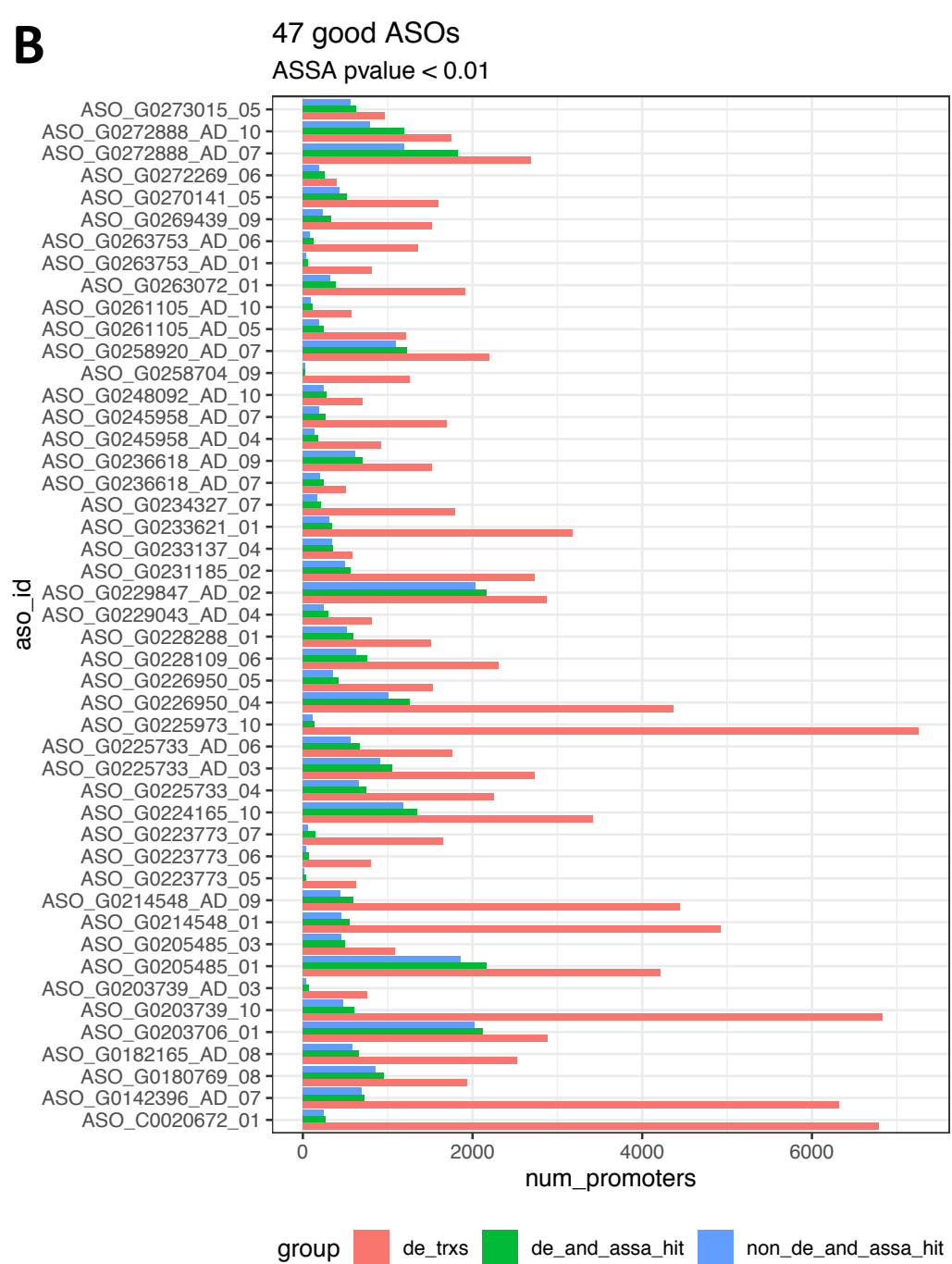

Supplement: Supplementary file 1 [file genes-11-01483-s001.zip › Supplementary Data S1/Supplementary Data/images/assa_hits_among_de_and_non_de.pdf]

# 47 good ASOs

ASSA pvalue < 0.01

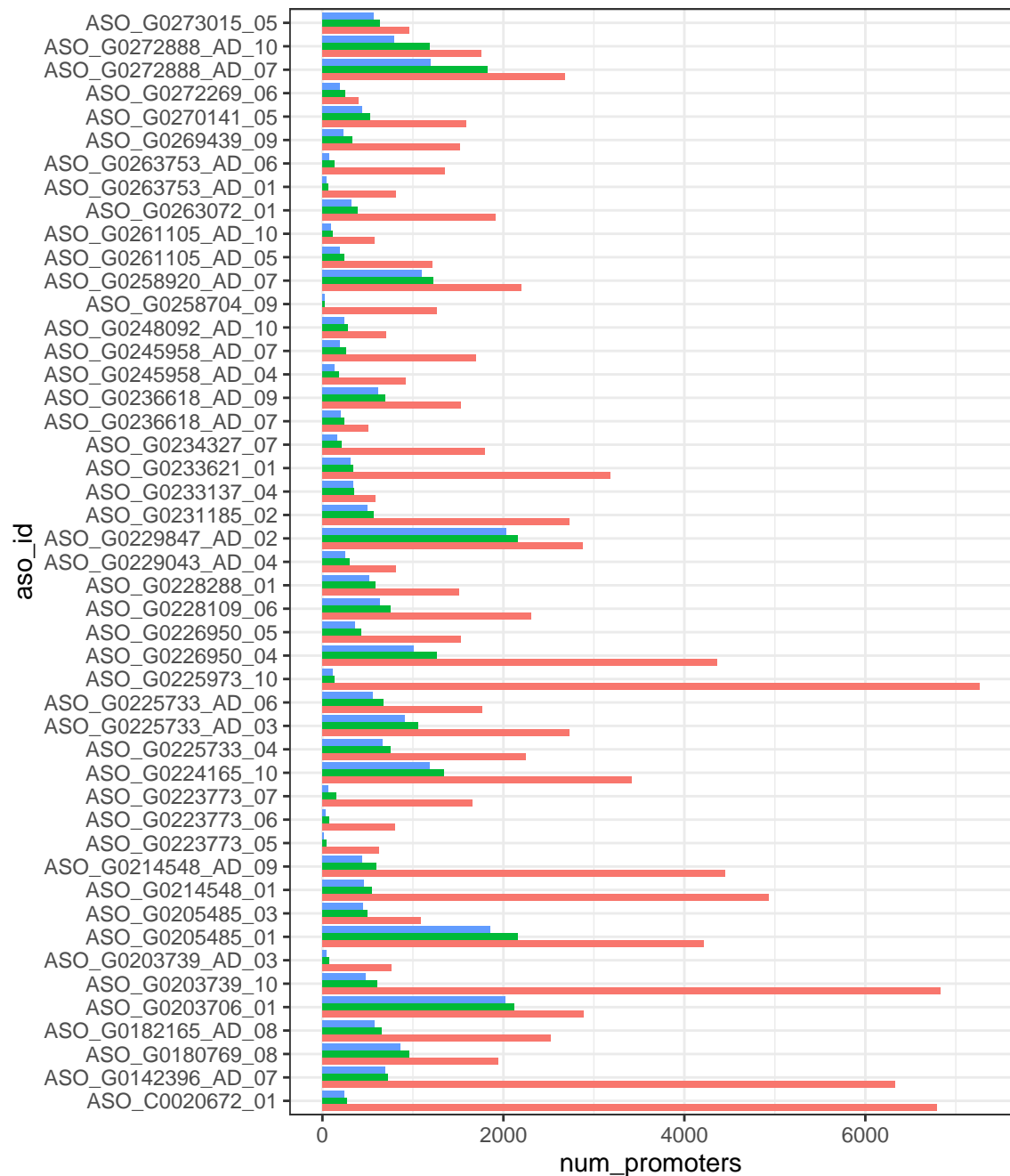

group

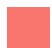

de\_trxs

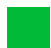

de\_and\_assa\_hit

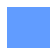

non\_de\_and\_assa\_hit

Supplement: Supplementary file 1 [file genes-11-01483-s001.zip › Supplementary Data S1/Supplementary Data/images/assa_hits_among_de_and_non_de.POST.pdf]

## Slide 1
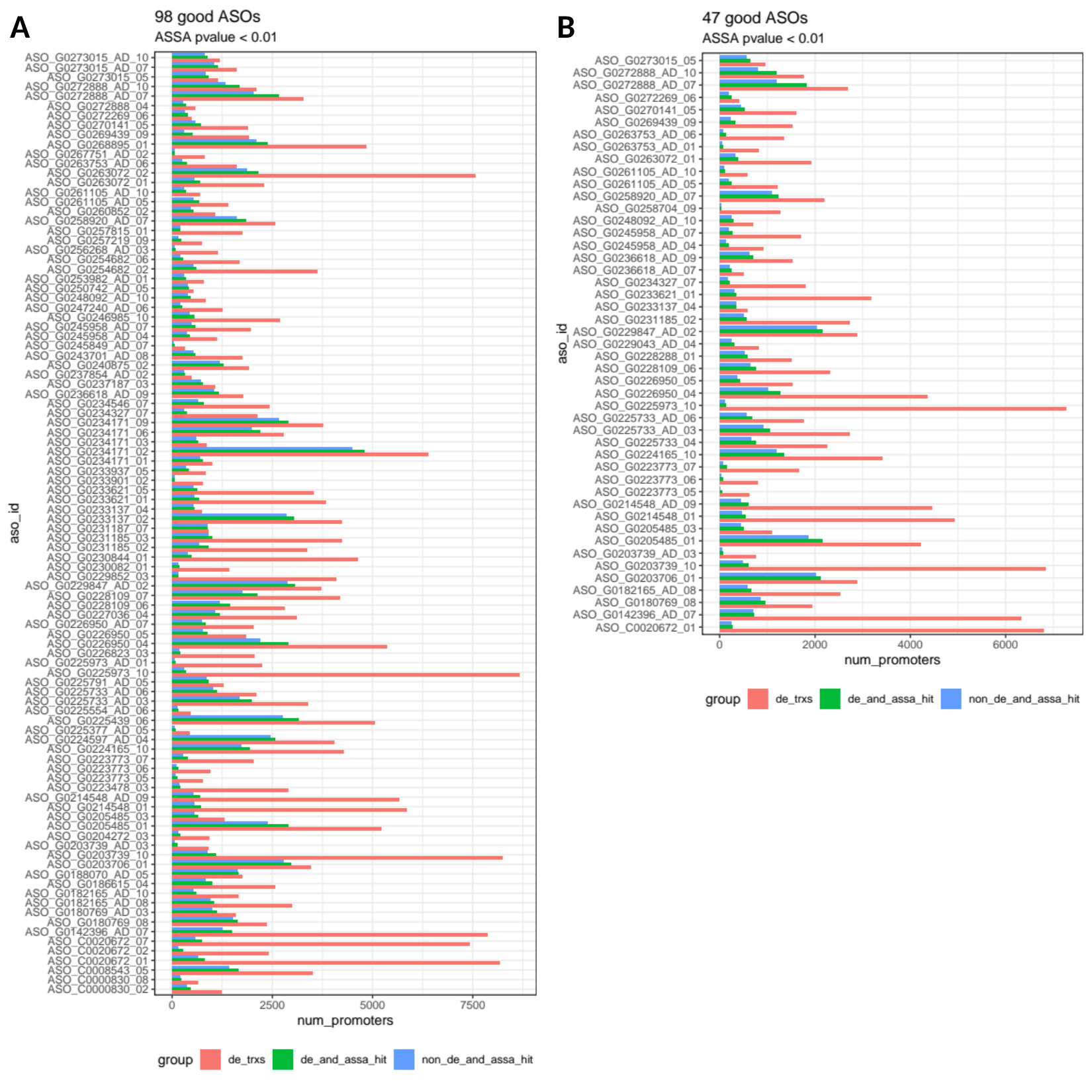

B
A

Supplement: Supplementary file 1 [file genes-11-01483-s001.zip › Supplementary Data S1/Supplementary Data/images/assa_hits_among_de_and_non_de.pptx]

**A**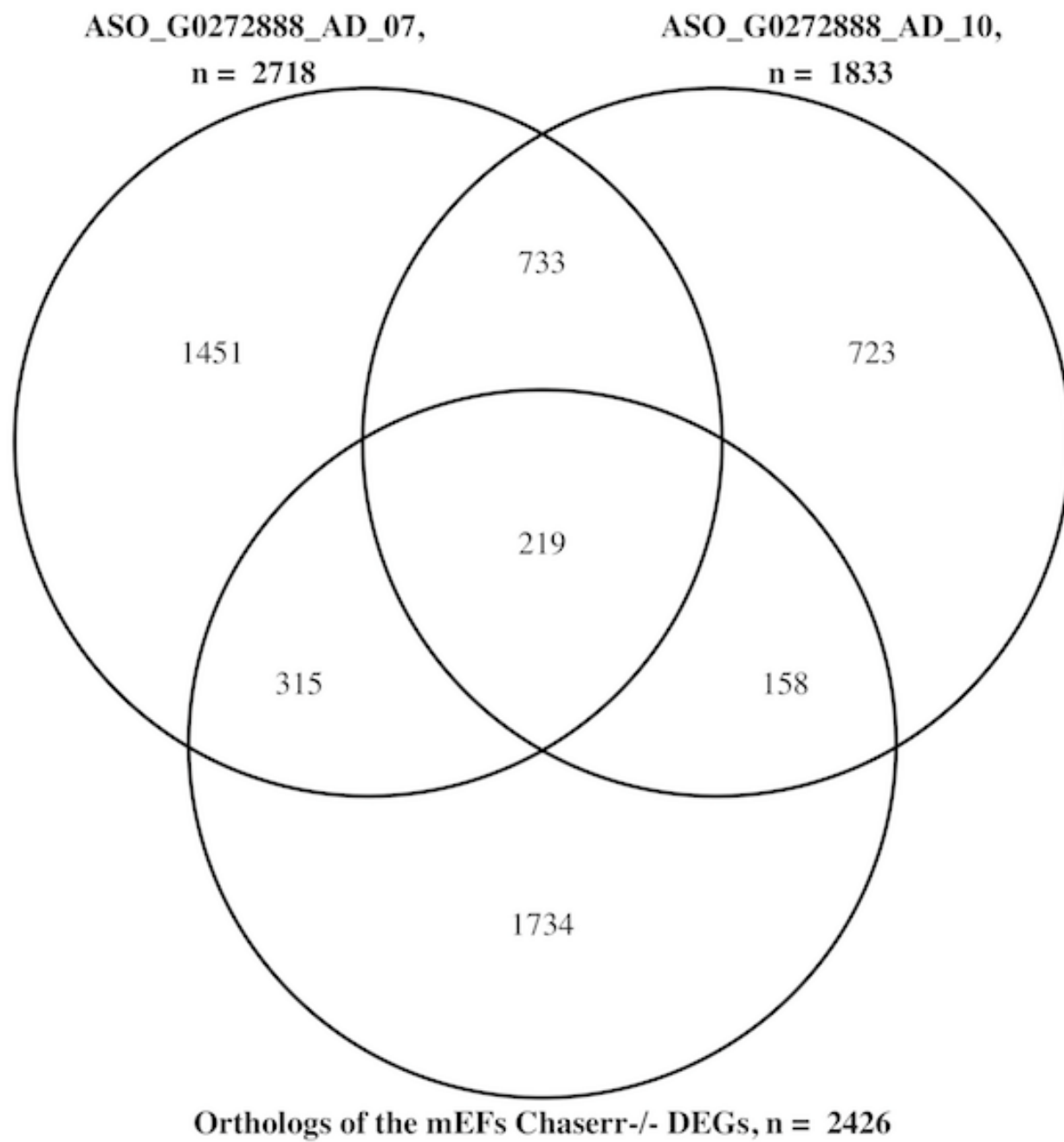**B**

219 common target genes

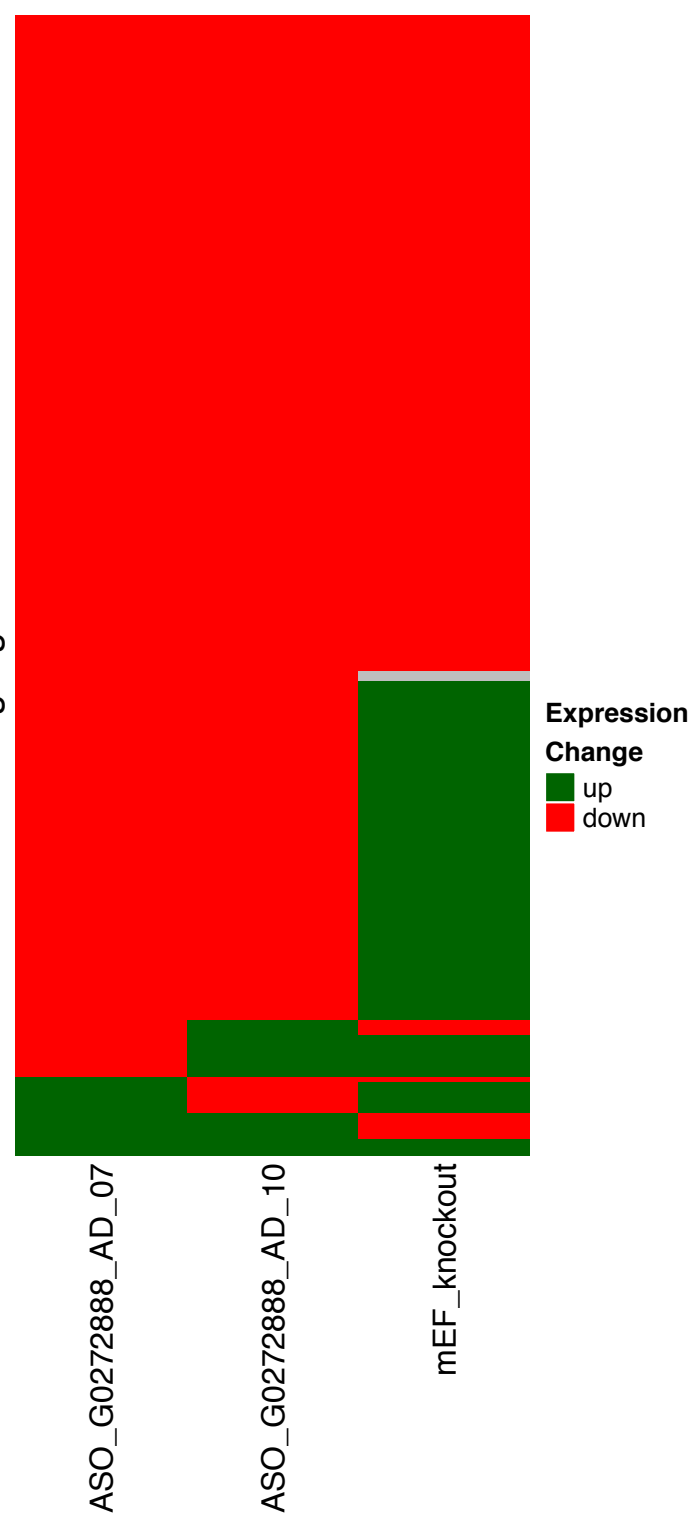

Supplement: Supplementary file 1 [file genes-11-01483-s001.zip › Supplementary Data S1/Supplementary Data/images/chaserr.pdf]

## Slide 1
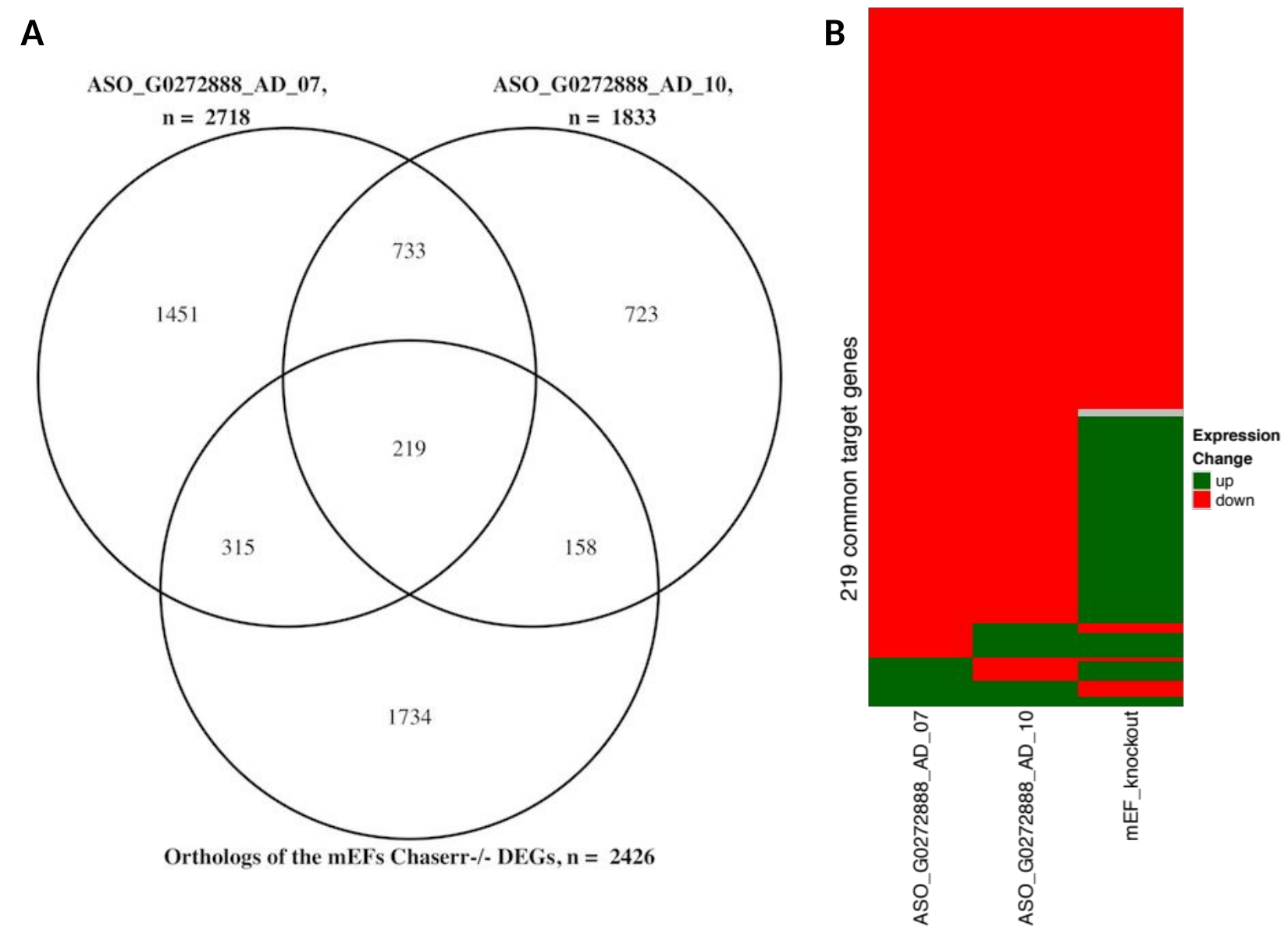

B
A

Supplement: Supplementary file 1 [file genes-11-01483-s001.zip › Supplementary Data S1/Supplementary Data/images/chaserr.pptx]

## Slide 1
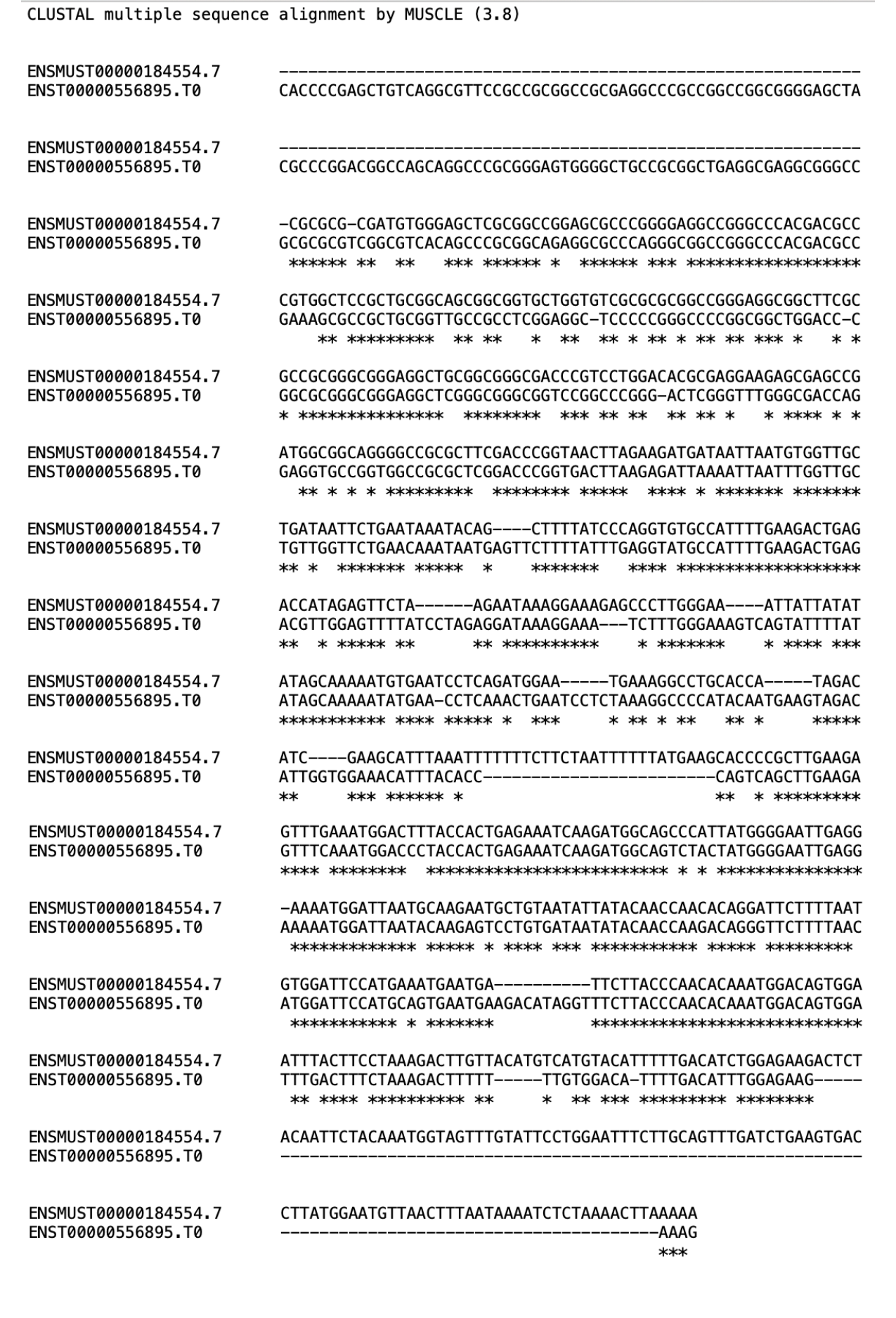

Supplement: Supplementary file 1 [file genes-11-01483-s001.zip › Supplementary Data S1/Supplementary Data/images/chaserr_alignment.pptx]

Expression change ■ up ■ down

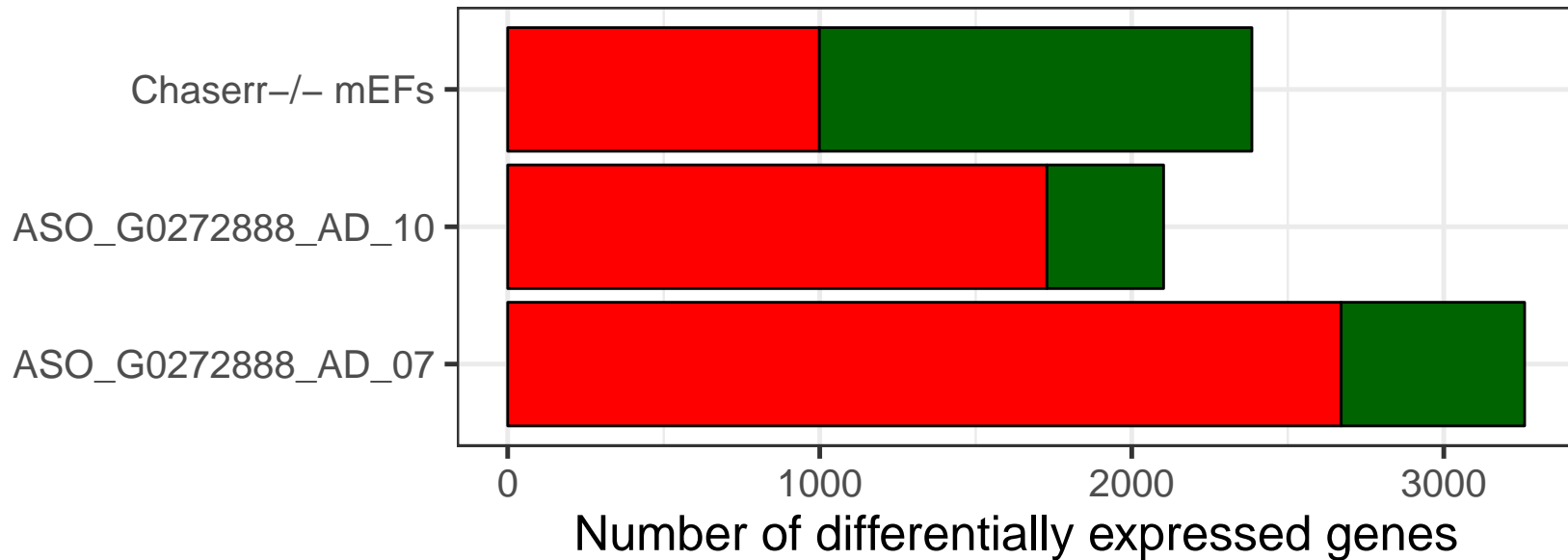

Supplement: Supplementary file 1 [file genes-11-01483-s001.zip › Supplementary Data S1/Supplementary Data/images/chaserr_up_down.pdf]

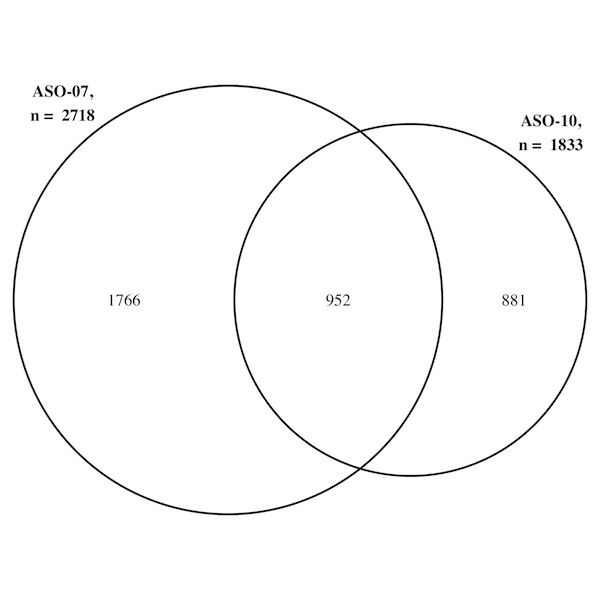

Supplement: Supplementary file 1 [file genes-11-01483-s001.zip › Supplementary Data S1/Supplementary Data/images/chaserr_venn_aso.png]

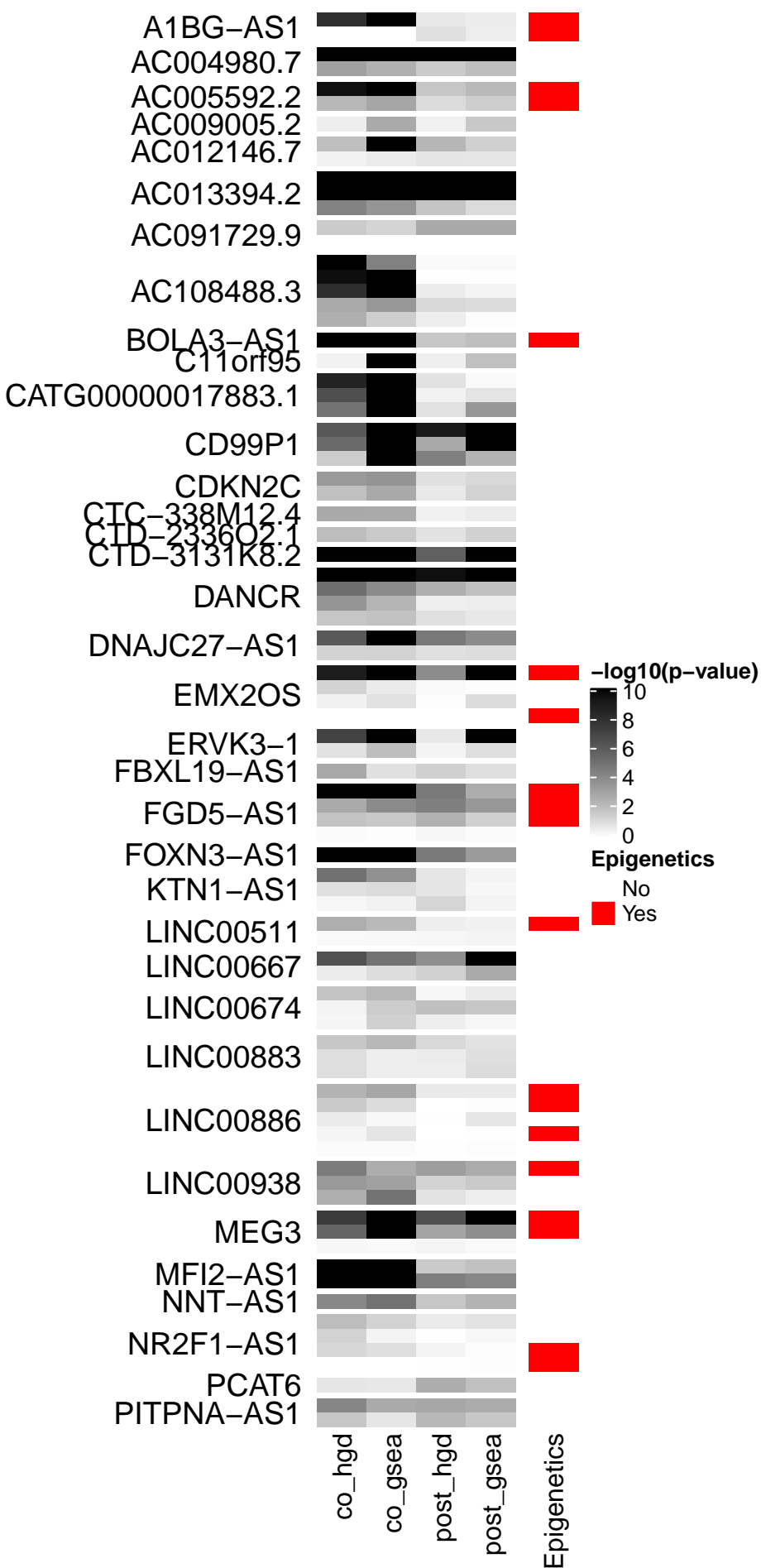

Supplement: Supplementary file 1 [file genes-11-01483-s001.zip › Supplementary Data S1/Supplementary Data/images/heatmap_4_pvalues_1.pdf]

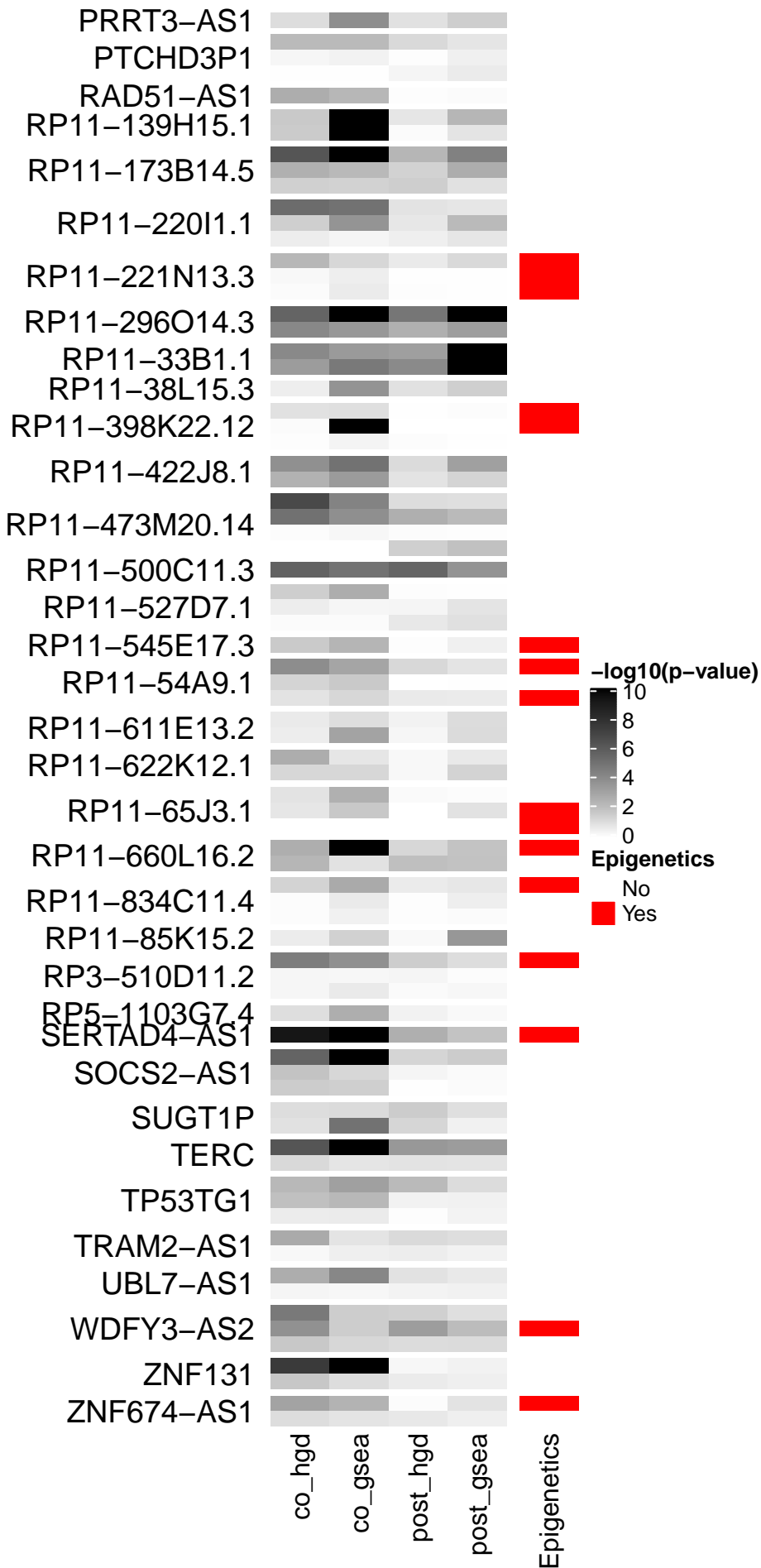

Supplement: Supplementary file 1 [file genes-11-01483-s001.zip › Supplementary Data S1/Supplementary Data/images/heatmap_4_pvalues_2.pdf]

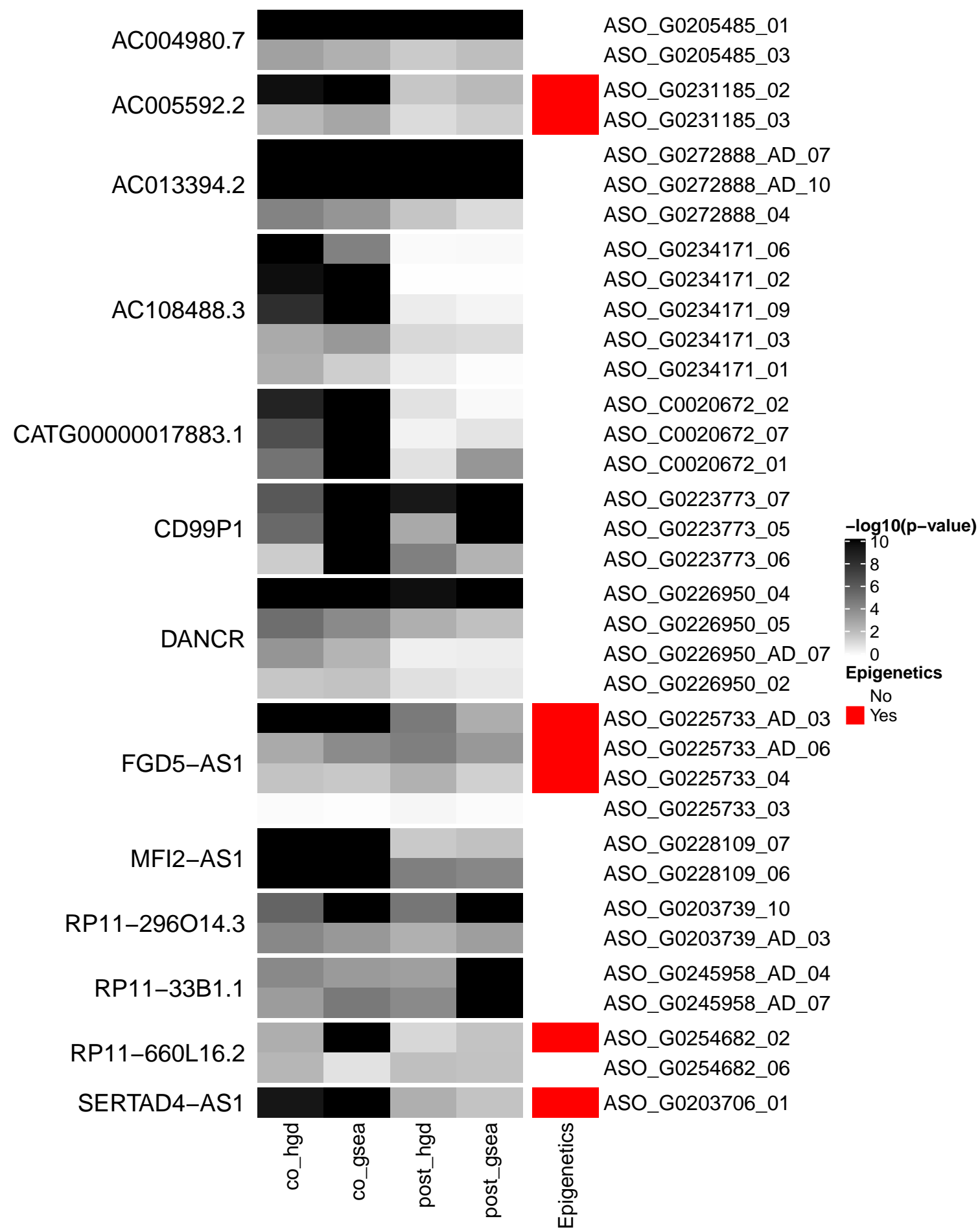

Supplement: Supplementary file 1 [file genes-11-01483-s001.zip › Supplementary Data S1/Supplementary Data/images/heatmap_4_pvalues_selected.pdf]

## Slide 1
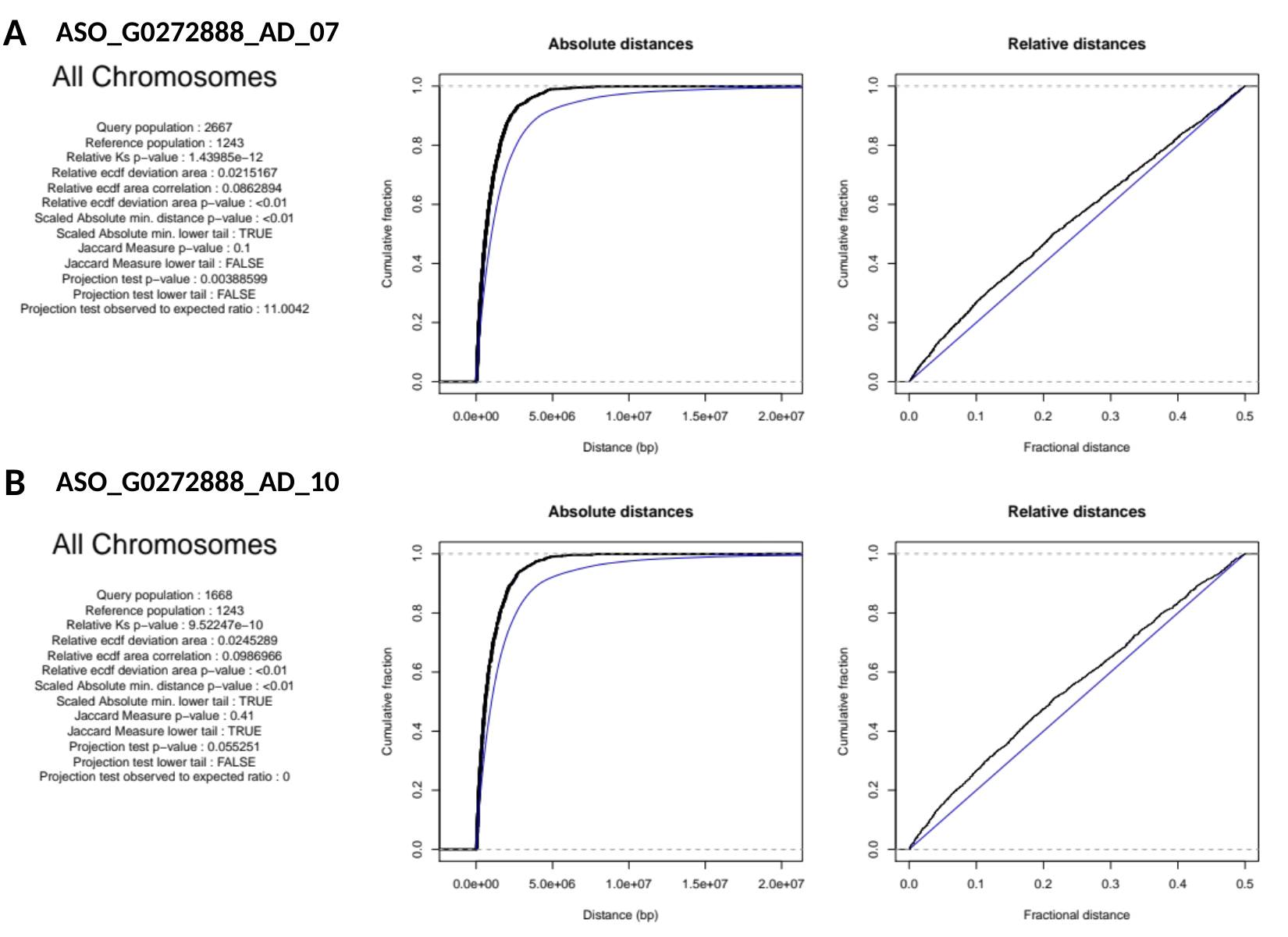

A
ASO_G0272888_AD_07
B
ASO_G0272888_AD_10

Supplement: Supplementary file 1 [file genes-11-01483-s001.zip › Supplementary Data S1/Supplementary Data/images/margi_genometric.pptx]

CHASERR  
(AC013394.2)  
lncRNA

CHD2

high gene  
expression

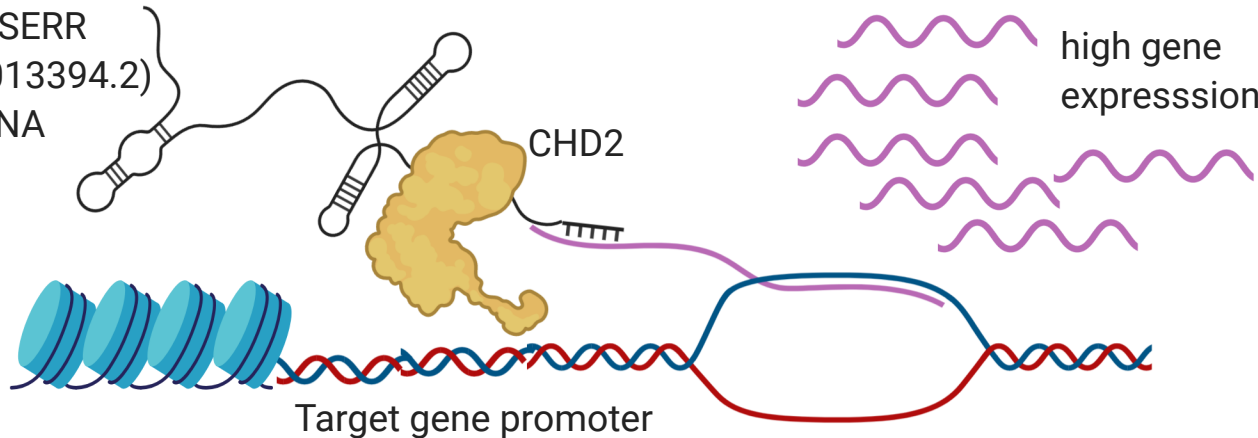

CHASERR  
knockdown /  
knockout

low gene  
expression

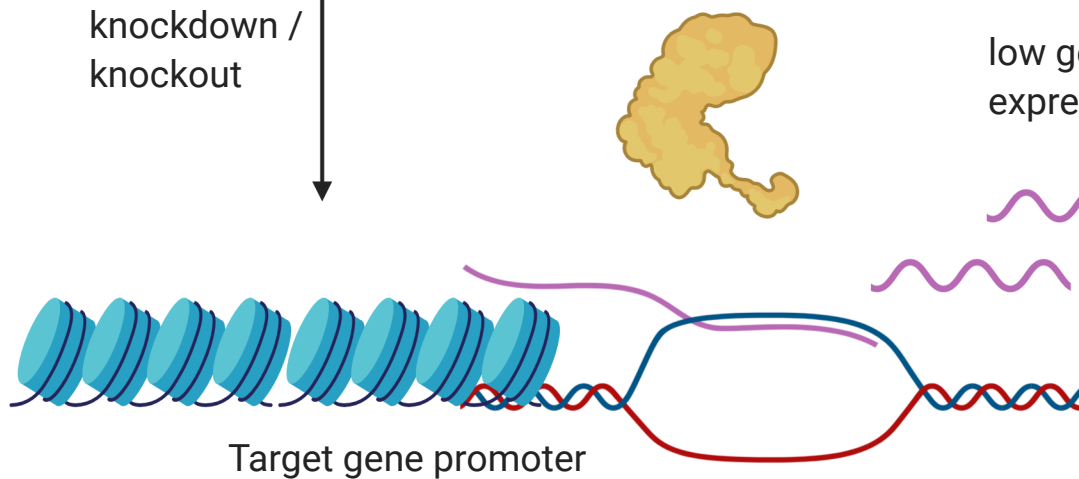

Supplement: Supplementary file 1 [file genes-11-01483-s001.zip › Supplementary Data S1/Supplementary Data/images/model.pdf]

## 219 common target genes

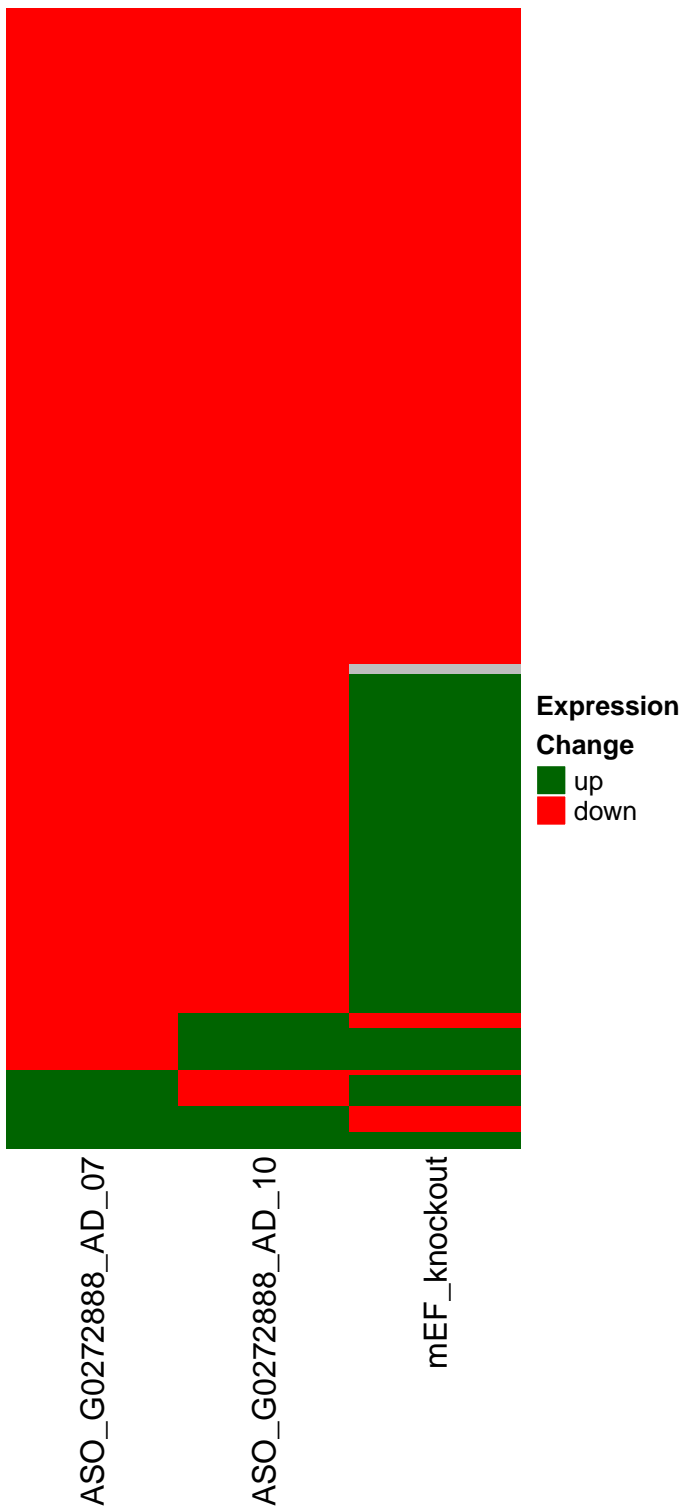

Supplement: Supplementary file 1 [file genes-11-01483-s001.zip › Supplementary Data S1/Supplementary Data/images/other/chaserr_common_genes_ht.pdf]

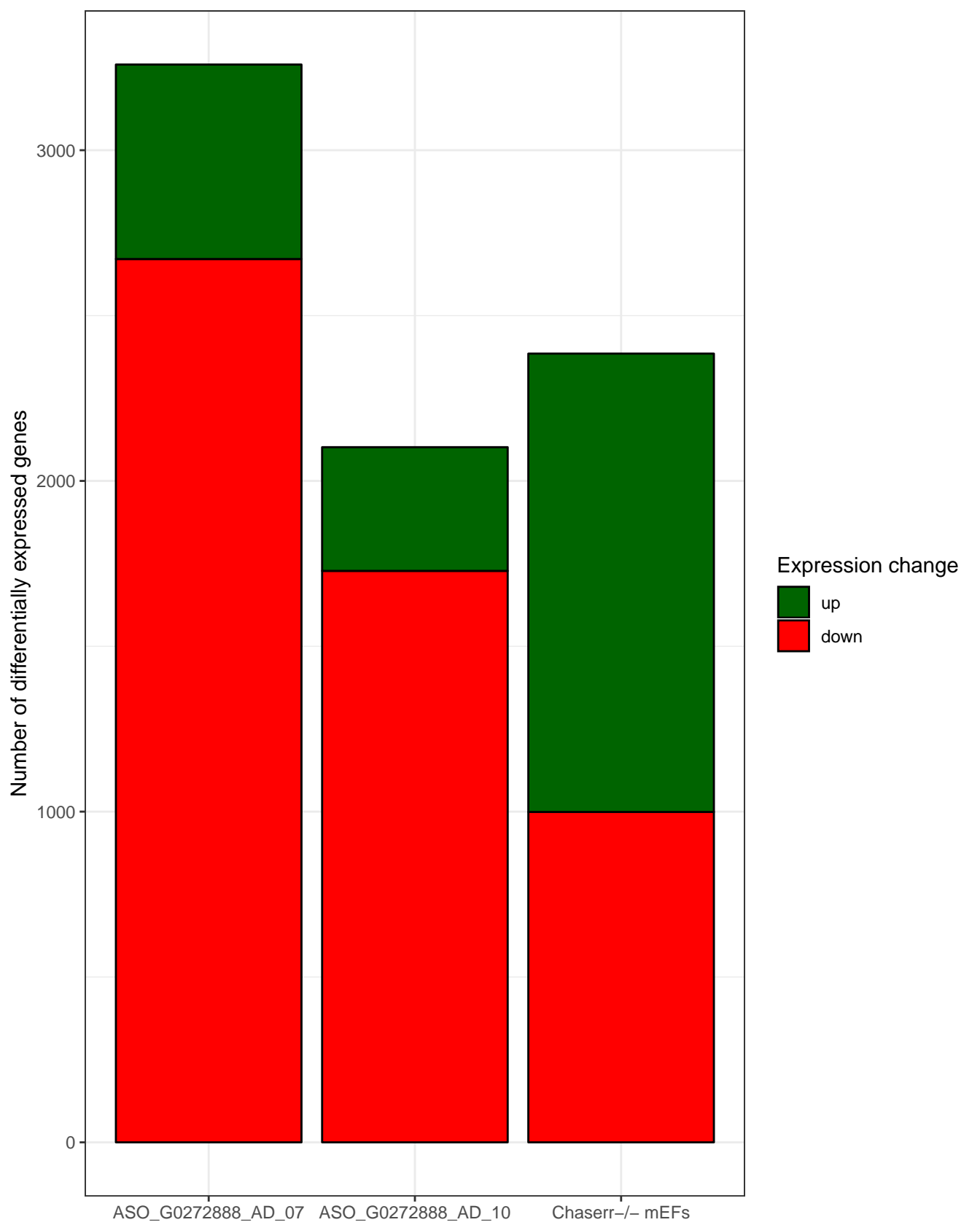

Supplement: Supplementary file 1 [file genes-11-01483-s001.zip › Supplementary Data S1/Supplementary Data/images/other/chaserr_up_down.pdf]

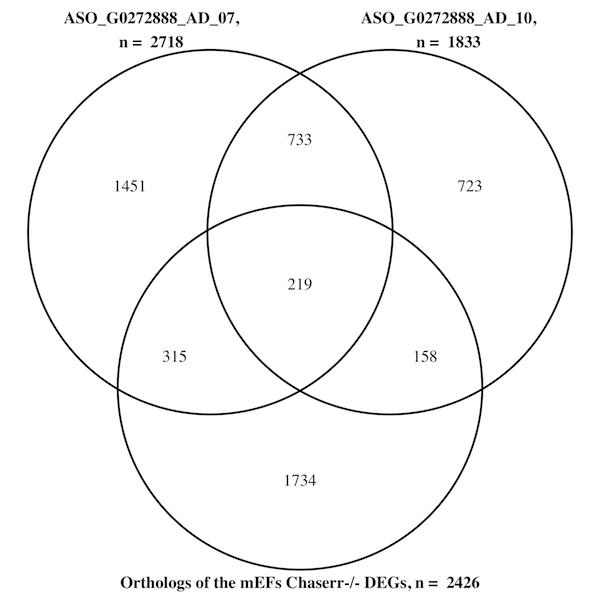

Supplement: Supplementary file 1 [file genes-11-01483-s001.zip › Supplementary Data S1/Supplementary Data/images/other/chaserr_venn_3.tiff]

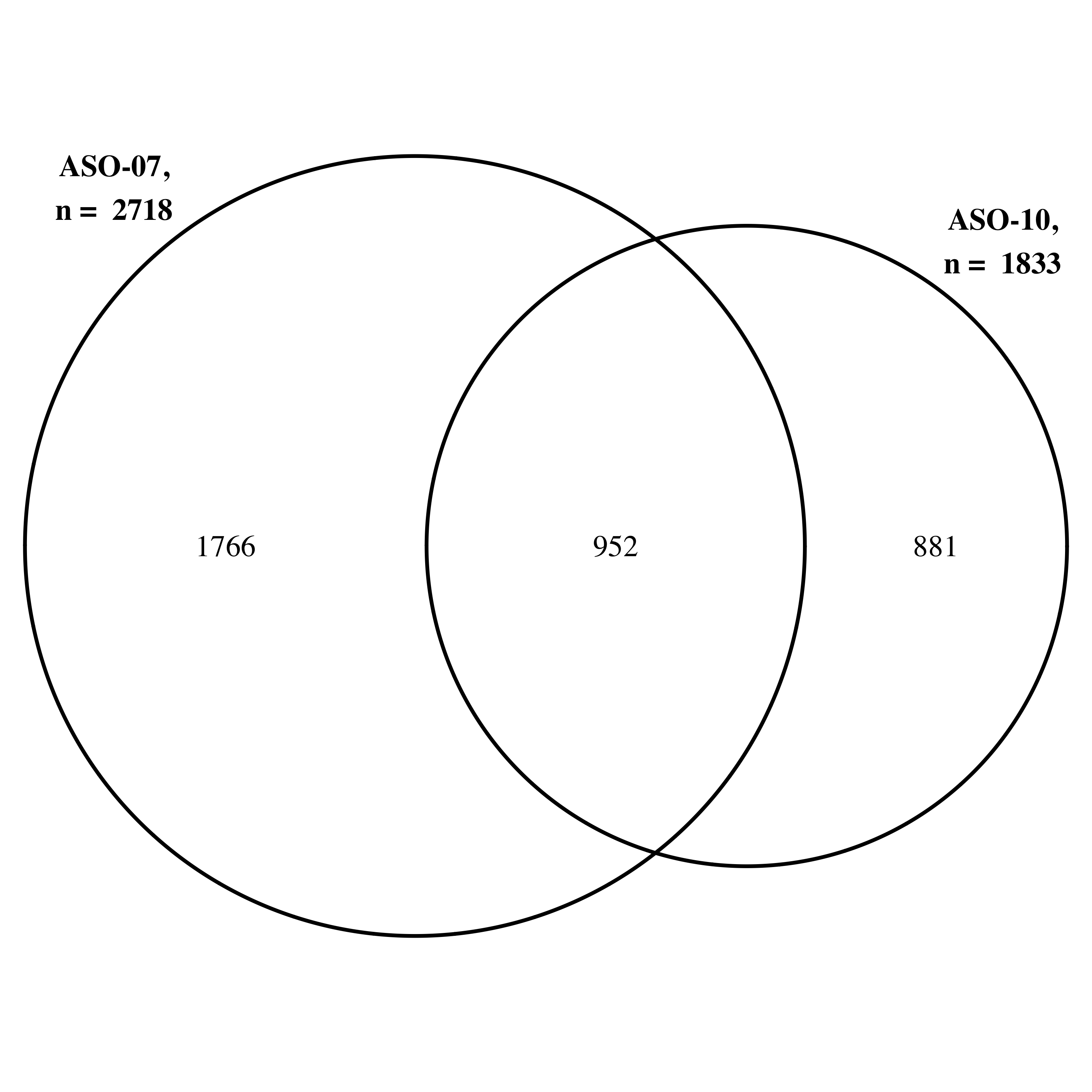

Supplement: Supplementary file 1 [file genes-11-01483-s001.zip › Supplementary Data S1/Supplementary Data/images/other/chaserr_venn_aso.png]

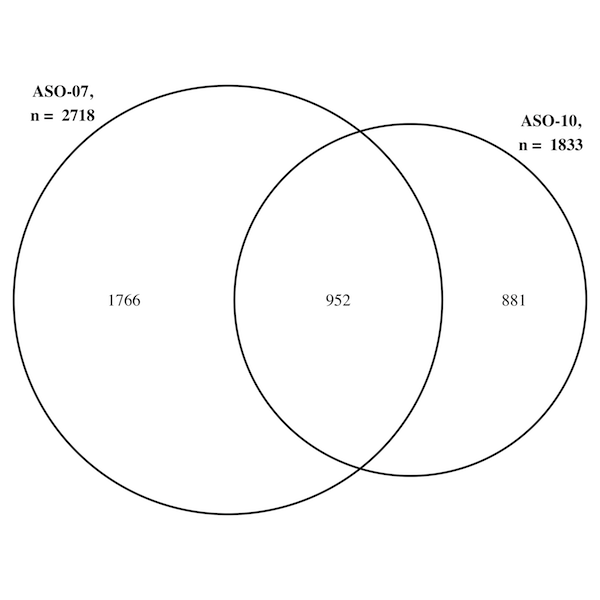

Supplement: Supplementary file 1 [file genes-11-01483-s001.zip › Supplementary Data S1/Supplementary Data/images/other/chaserr_venn_aso.tiff]

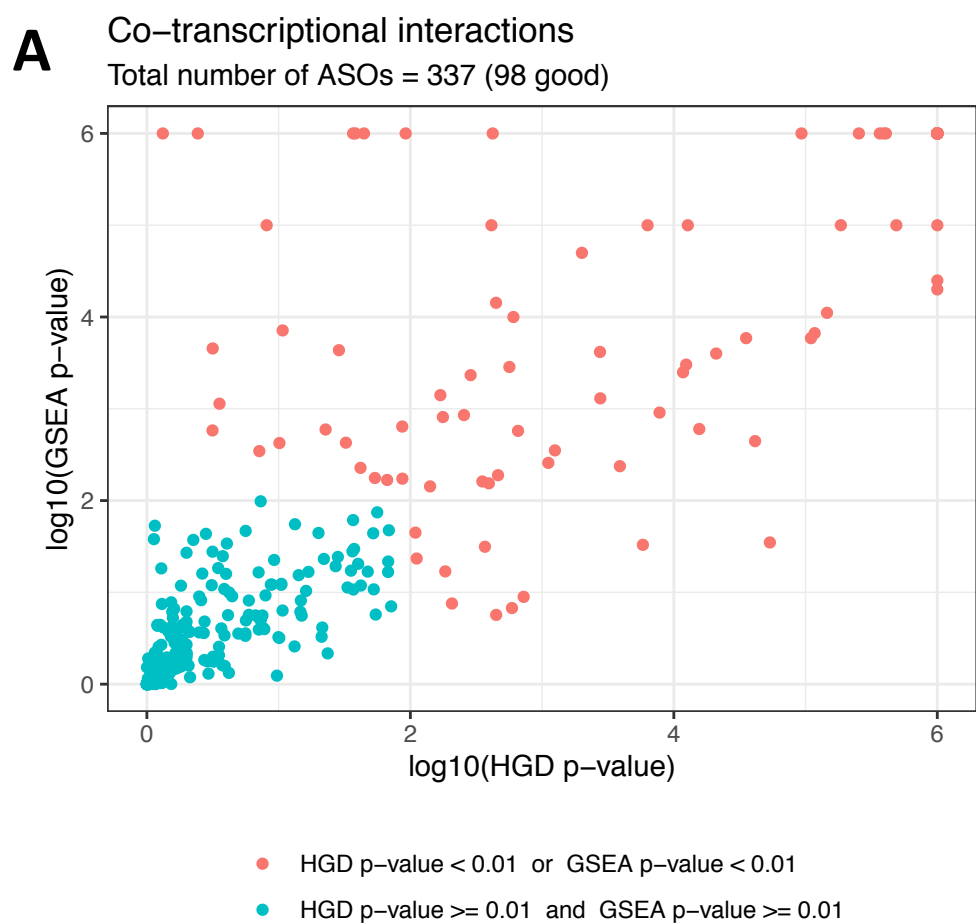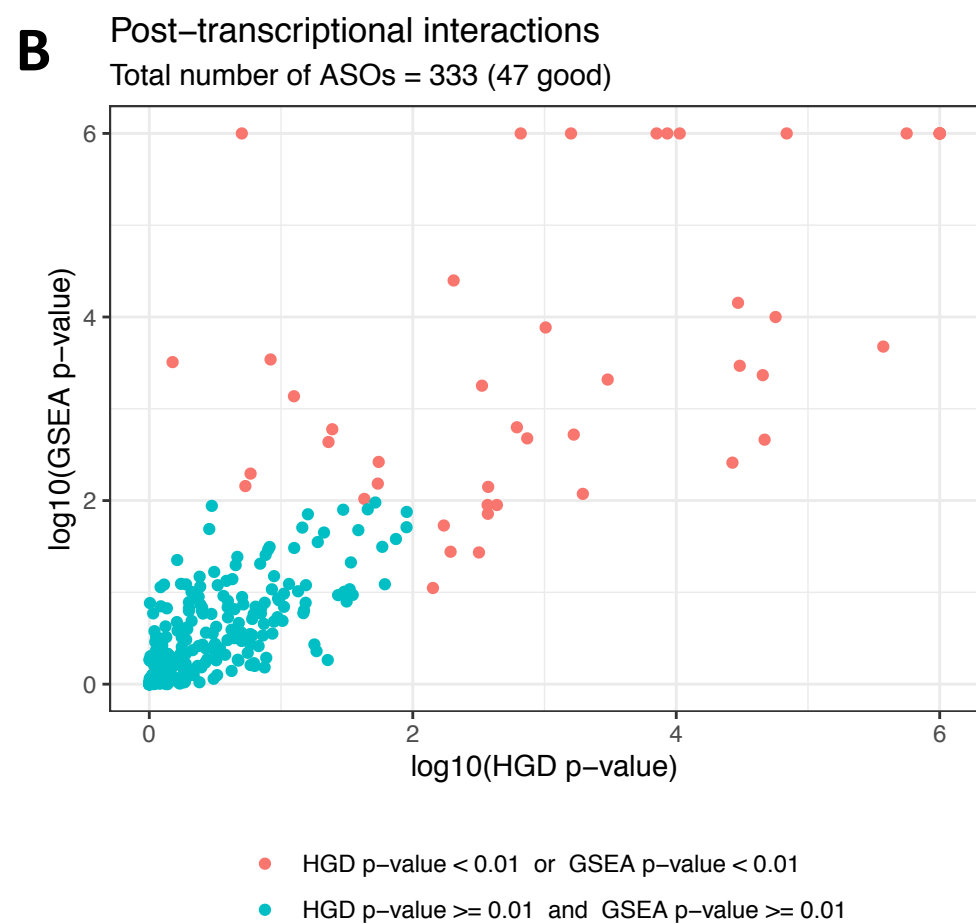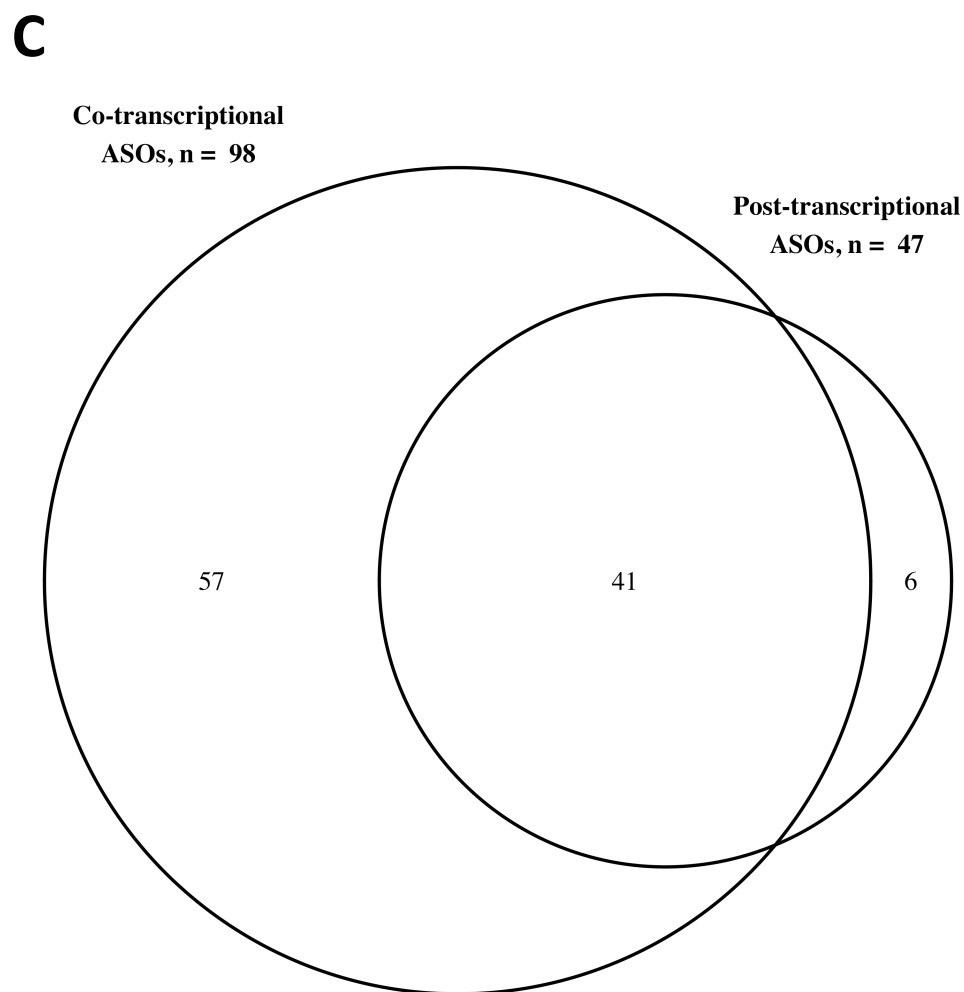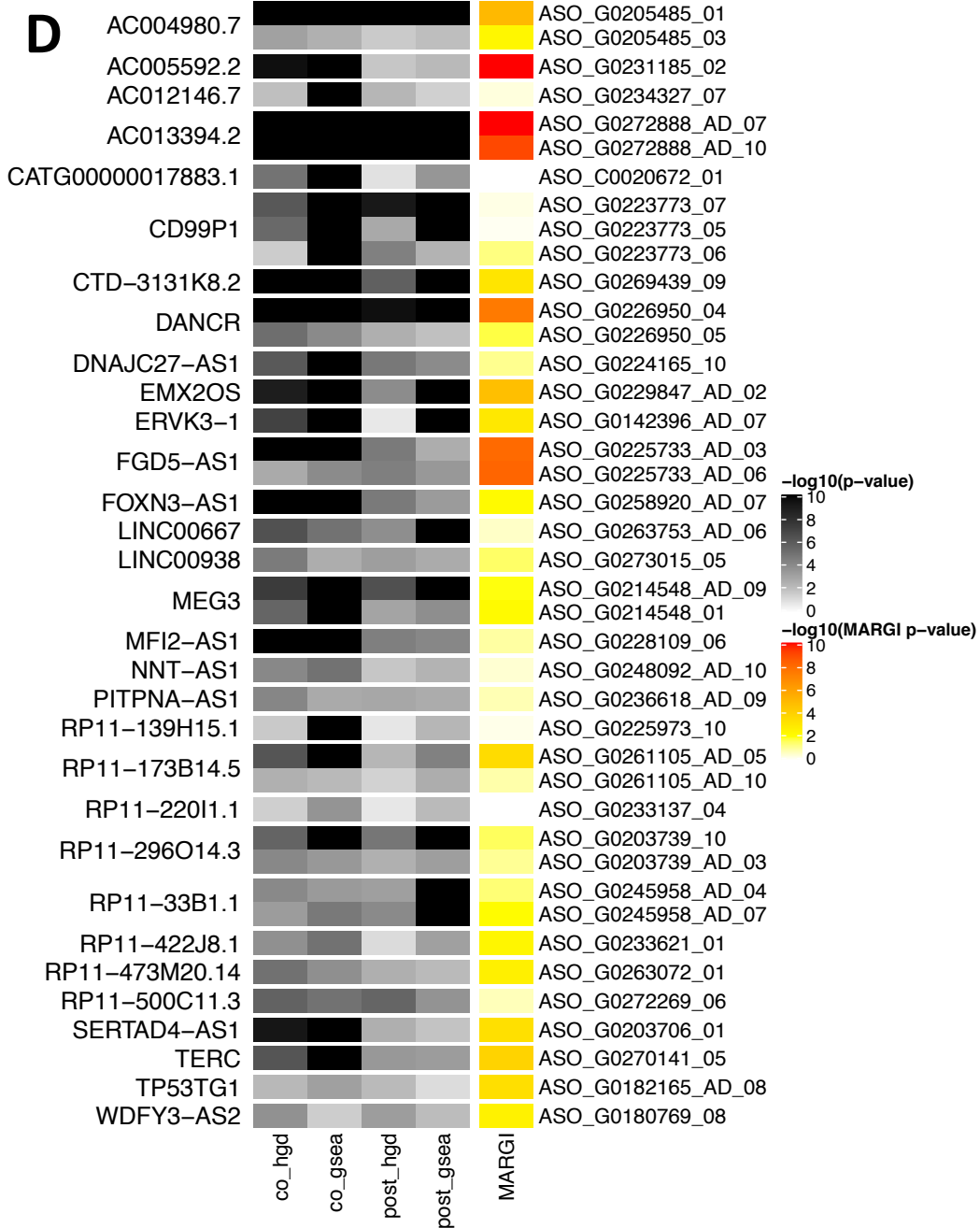

Supplement: Supplementary file 1 [file genes-11-01483-s001.zip › Supplementary Data S1/Supplementary Data/images/two_pvalues.pdf]

## Slide 1
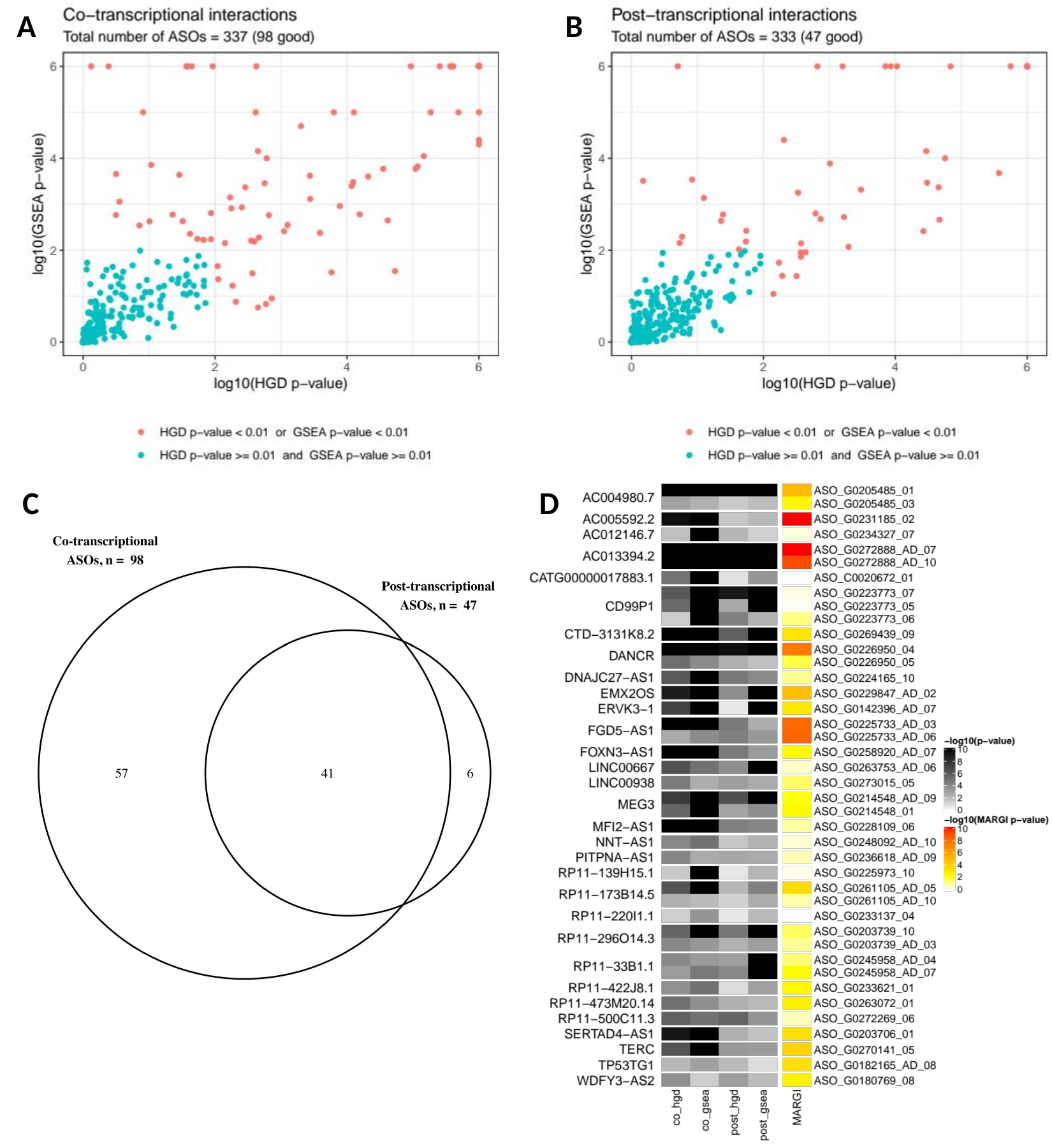

B
A
C
D

Supplement: Supplementary file 1 [file genes-11-01483-s001.zip › Supplementary Data S1/Supplementary Data/images/two_pvalues.pptx]

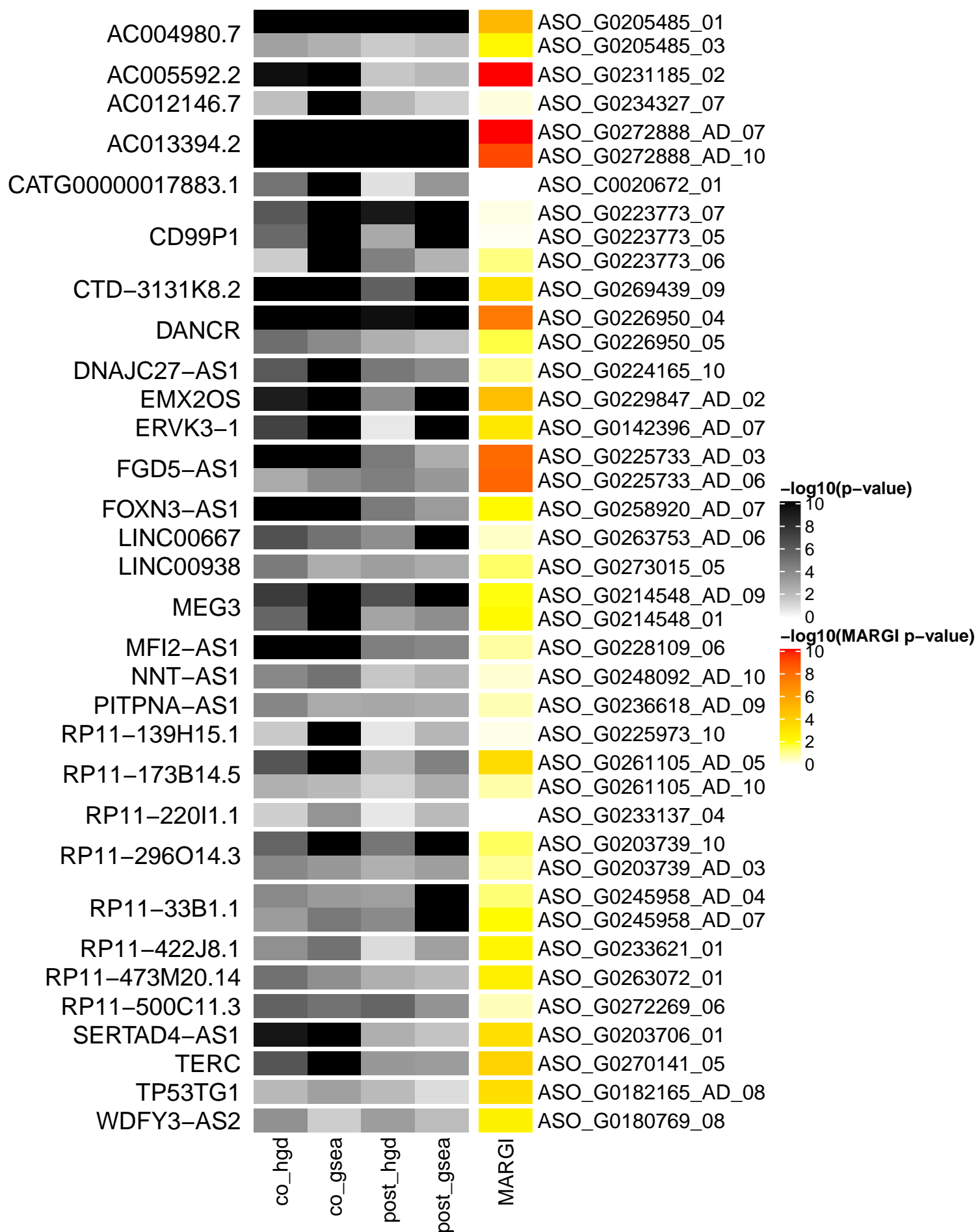

Supplement: Supplementary file 1 [file genes-11-01483-s001.zip › Supplementary Data S1/Supplementary Data/images/two_pvalues_heatmap.pdf]

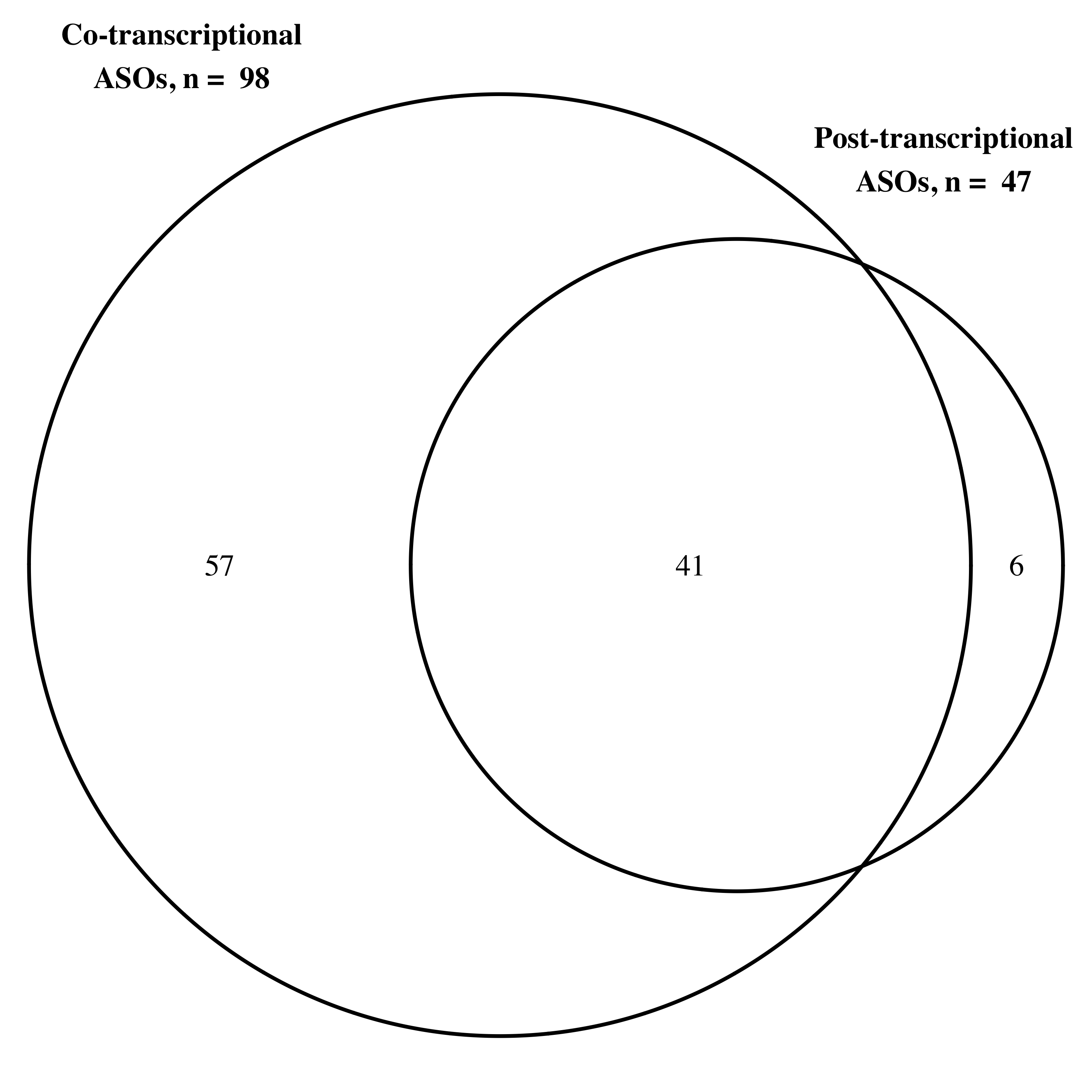

Supplement: Supplementary file 1 [file genes-11-01483-s001.zip › Supplementary Data S1/Supplementary Data/images/two_pvalues_venn.png]

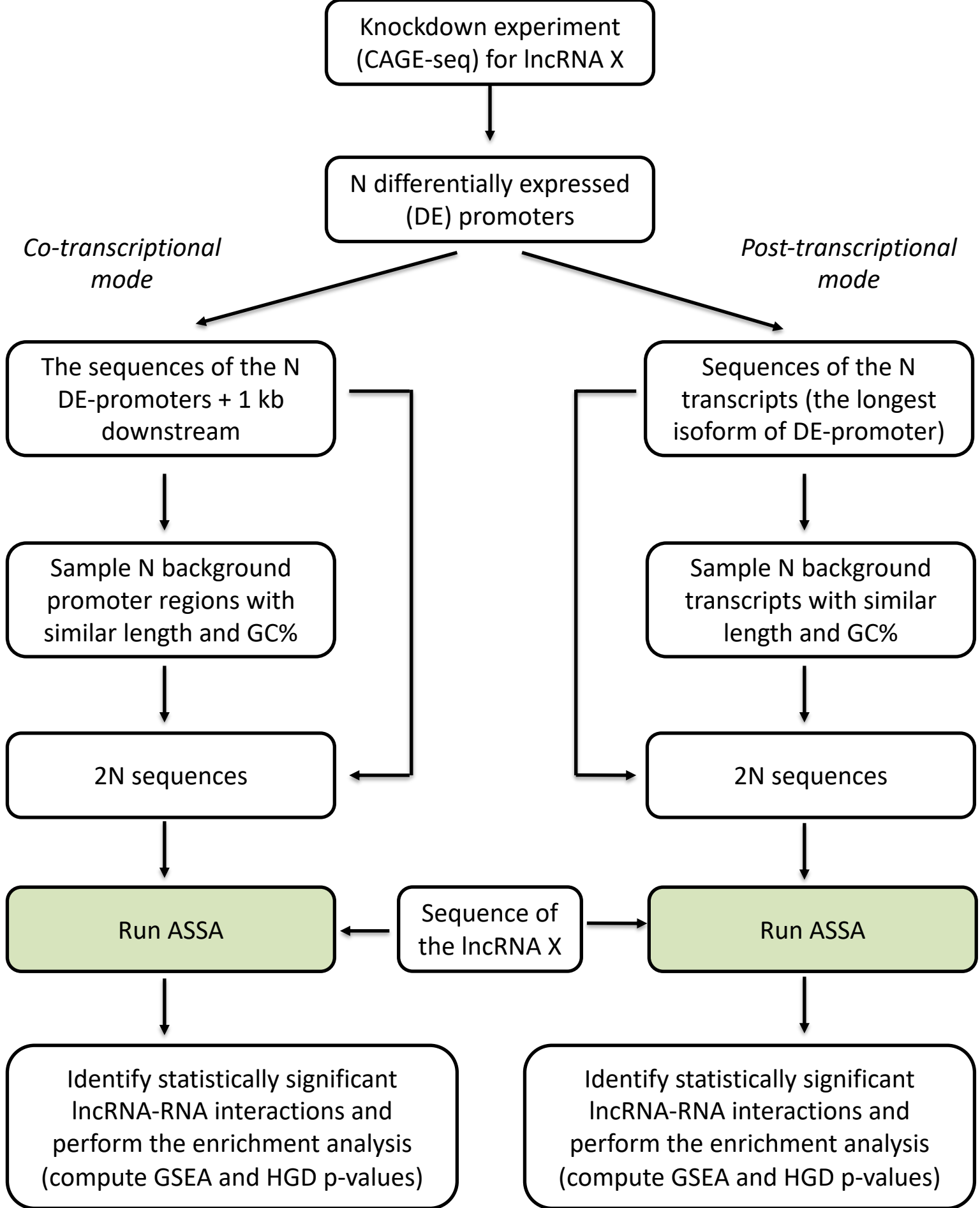

Supplement: Supplementary file 1 [file genes-11-01483-s001.zip › Supplementary Data S1/Supplementary Data/images/workflow.pdf]
